# Gene expression profiling of chicken primordial germ cell ESTs

**DOI:** 10.1186/1471-2164-7-220

**Published:** 2006-08-30

**Authors:** Jae Yong Han, Tae Sub Park, Jin Nam Kim, Mi A Kim, Dajeong Lim, Jeong Mook Lim, Heebal Kim

**Affiliations:** 1 Department of Food and Animal Biotechnology, Seoul National University, Seoul 151-921, Korea; 2 Avicore Biotechnology Institute Inc., Hanlim Human Tower #707, Geumjeong-dong 1-40, Gunpo city, Gyeonggi-do 435-050, Korea

## Abstract

**Background:**

Germ cells are the only cell type that can penetrate from one generation to next generation. At the early embryonic developmental stages, germ cells originally stem from primordial germ cells, and finally differentiate into functional gametes, sperm in male or oocyte in female, after sexual maturity. This study was conducted to investigate a large-scale expressed sequence tag (EST) analysis in chicken PGCs and compare the expression of the PGC ESTs with that of embryonic gonad.

**Results:**

We constructed 10,851 ESTs from a chicken cDNA library of a collection of highly separated embryonic PGCs. After chimeric and problematic sequences were filtered out using the chicken genomic sequences, there were 5,093 resulting unique sequences consisting of 156 contigs and 4,937 singlets. Pearson chi-square tests of gene ontology terms in the 2nd level between PGC and embryonic gonad set showed no significance. However, digital gene expression profiling using the Audic's test showed that there were 2 genes expressed significantly with higher number of transcripts in PGCs compared with the embryonic gonads set. On the other hand, 17 genes in embryonic gonads were up-regulated higher than those in the PGC set.

**Conclusion:**

Our results in this study contribute to knowledge of mining novel transcripts and genes involved in germline cell proliferation and differentiation at the early embryonic stages.

## Background

Primordial germ cells (PGCs), the precursor of gametes, have a unique migration activity in birds as well as in mammals. They temporally reside in the extra-embryonic tissue and localize into embryonic gonads. PGCs in mammals are originally derived from the epiblast of the gastrulating embryo and move into embryonic gonads through hindgut by amoeboid movement [1]. In contrast, in birds, PGCs firstly appear from the epiblast in the blastoderm and translocate to the hypoblast of the area pellucida [2,3]. During the gastrulation, they circulate through the vascular system and shuttle down into the gonadal anlagen [1]. Thus, avian PGCs can be collected from germinal crescent [4,5] or blood vessel [6,7], and embryonic gonads [8-10]. Recently, this unique migration pattern of avian PGCs allowed producing germline chimeras by re-transplantation of the PGCs into the blood vessel of recipient embryos [9-11].

Morphological and physiological features of avian germ cells including PGCs have been well characterized and utilized for further studies. However, there are only a few reports on expressed sequence tag (EST) analysis and functional genomic study for avian germ cells, especially PGCs in the early embryonic developmental stages. PGC is an important cell type, in which either gene expression or suppression should be regulated temporally and spatially during embryonic developments. According to gene expression switching triggered by interactions with environmental niche, PGCs could maintain their pluripotency or differentiate into germ cells. However, due to technical difficulties, no further progress has been made in functional genomic study and massive novel gene mining in avian PGCs as well as neighboring stroma cells.

Therefore, this study was conducted to investigate a large-scale EST analysis in chicken MACS-separated PGCs and compared the expression of the PGC ESTs with that of embryonic gonad.

## Results and discussion

### Retrieval of chicken PGCs by MACS treatment

Embryonic gonads were retrieved from total 7,955 White Leghorn (WL) embryos at 6.5 days of incubation and then the retrieved gonadal cells were treated with magnetic activated cell sorter (MACS) for separation of PGCs in total embryonic gonadal cells. PGC population ratio after MACS separation increased 47.4 folds than that before MACS (35.1% vs. 0.74%). Thus, we collected 7.7 × 10^6 ^PGCs from 7,955 embryos for total RNA preparation and cDNA library construction. The population ratio of chicken PGCs in embryonic gonads was approximately 0.74% [12]. So, it is difficulty and complicated to retrieve a large number of chicken PGCs at the early embryonic stages. Thus, in this study, gene expression profiling of chicken PGCs was conducted and analyzed with MACS-separated chicken PGCs. The morphological and physiological properties of PGCs were unchanged even after MACS separation [12]. MACS-separated PGCs has the reactivity to germ cell-specific antibodies and the migration capacity into embryonic gonads after re-transplantation into the recipient embryos [12].

### cDNA library construction from chicken PGCs and EST sequencing

After *in vivo *excision with *E. coli *strain SOLR, insert sizes of the cDNA libraries from PGCs were analyzed by PCR and insert fragments ranged from 0.5 to 3 kb (n = 18). Titer of primarily cDNA library was approximately 4.0 × 10^6 ^pfu/ml on average. Subsequently, we massively sequenced ESTs from PGC cDNA library and total 10,944 ESTs were sequenced. Of 10,944 ESTs, 96 sequences were excluded due to low sequencing quality and after vector sequence trimming. Thus, finally 10,848 were used for computational analysis.

### ESTs processing and assembling

We have sequenced 10,851 cDNA clones from MACS-separated chicken PGC population cDNA library generating 10,848 sequences. The EST data that are described in this paper have been submitted to the NCBI dbEST under accession nos. DR410159-DR421006. Assembling and clustering of the EST data resulted in 8,914 unique sequences with 242 contigs and 7,196 singlets. Filtering out possible chimeric sequences with similarity search against chicken genomic sequences were removed 86 contigs and 2,259 singlets resulting in total 5,093 sequences with 156 contigs and 4,937 singlets. Since the genomic sequences do not have 100 % coverage and accuracy, some of the filtered sequences might be genuine sequences but did not correspond with the genomic sequence draft. Average number of ESTs per contig was about 3.9. On the other hands, clustering and assembling of embryonic gonad ESTs (NCBI dbEST accession nos. CV852525-CV862818) were resulted in total 5,751 unique sequences; 971 contigs and 4,780 singlets, respectively. Average EST numbers per a contig were approximately 4.1 in assemblies of embryonic gonad ESTs. The unique sequences of the two different sets described in this paper are available at Chicken Primordial Germ Cell ESTs [13].

### Gene ontology annotation and putative novel transcripts

**Figure 1:**
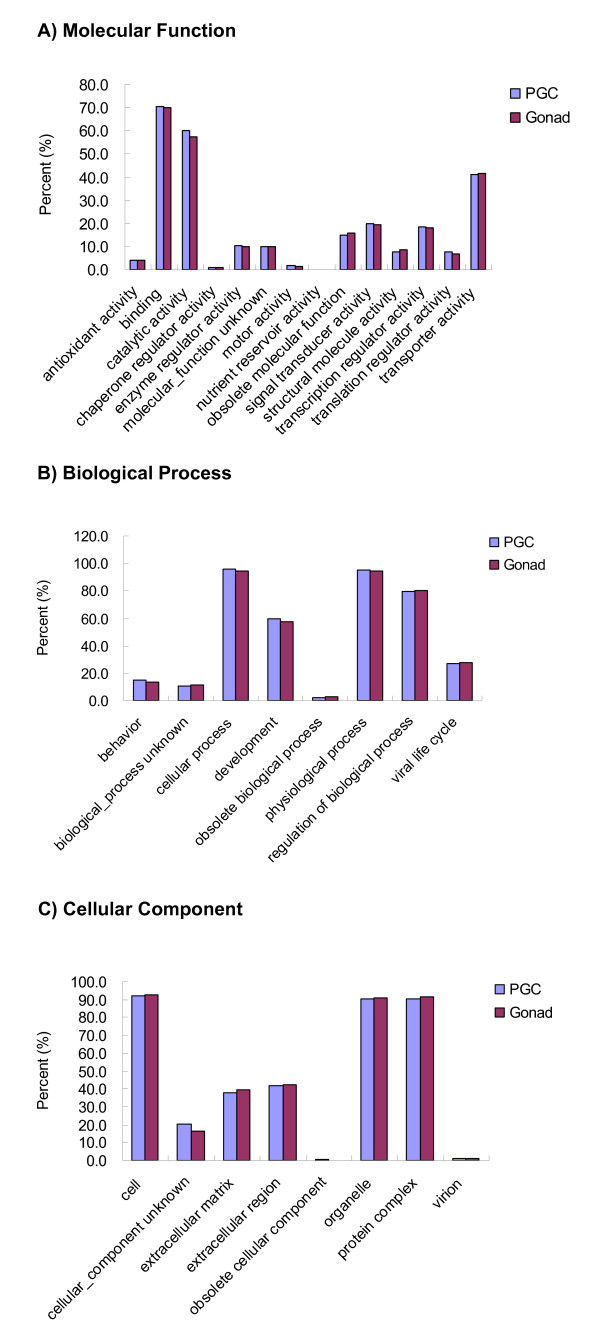
Percentage distributions of 2^nd ^level gene ontology terms. There were no significant p-values between primordial germ cells (PGCs) and embryonic gonad dataset in all of gene ontology terms when analyzed by using Pearson's chi-square test of independent. Overall significance level of alpha was given by the Bonferroni correction, alpha = 0.5/m, where m was 60 since 30 GO terms were compared between PGCs and embryonic gonad dataset.

Figure 1 shows percentage distributions of gene ontology terms, 2^nd ^level GO terms according to the GO consortium, of the two sets of non-redundant sequences. Pearson chi-square tests of independence between PGCs versus embryonic gonad sets indicated that most of the 2^nd ^level terms did not show any significance. Since the MACS-separated PGC population was approximately 35.1% and ontology annotation was conducted non-redundantly, it might be anticipated that distribution of annotated genes in PGC population set would be similar to gonad set and show no significance. In further experiment, we will conduct large-scale EST analysis with highly purified PGC population for discovering informative and novel transcripts related to germ cells.

The number of putative novel transcripts obtained by comparing with the GgGI (Release 10.0) was total 1,815 sequences with 31 contigs and 1,784 singlets. As it was not a cross-species sequence comparison, strict criteria as described in the method were applied to the cut-off values. For the same reason, identifying the putative novel transcripts can be seen as non-strict criteria. The reason for applying the strict threshold was to avoid blast hit with paralogous sequences. Functional prediction and gene ontology distribution on the basis of sequence similarity search against non-redundant protein database of the NCBI are shown in Additional file 1 and Additional file 2a. Interestingly, Pearson chi-square test of independence of 2^nd ^level GO terms between PGCs and novel transcripts data set indicates that a large portion of cellular component unknown term in cellular components would be involved in gene expression of PGCs [see Additional file 2b]. In addition, there were higher portion(s) of behavior and development in biological process, and obsolete molecular function and transporter activity in the novel transcripts than those of PGC set.

### Digital gene expression profiling

While the comparison of the Pearson chi-square test of particular GO terms is based on non-redundant data sets, digital gene expression profiling is based upon quantitative differences between two different datasets. Using the Audic's test [14], digital gene expression profiling is showed in Table 1. There were 2 genes expressed significantly higher number of transcripts in PGCs compared with embryonic gonads set. On the contrary, 17 genes in embryonic gonads were up-regulated higher than those in PGC set (Table 1). NADH dehydrogenase subunit 1 gene, one of two PGC-highly expressed genes, was located in chicken mitochondrial genome and its product was partially related to sperm activity regulated by mitochondrial functions [15], but 40S ribosomal protein SA was not reported in germ cell expression yet. Interestingly, 40S ribosomal protein SA or 37LRP/p40 was closely associated to invasive and metastatic activity in cancer cells [16]. During the formation of the undifferentiated gonads, PGCs actively penetrate into gonadal epithelium through blood vessel. Even after settled down in embryonic gonads, they invade from cortical layer into medullar tissue by active migration activity [1]. Thus, it might be assumed that 40S ribosomal protein SA is involved in active PGC mobility. However, further study should be conducted to elucidate its function in PGCs.

Among 17 gonad-highly expressed genes, several genes such as calmodulin, eukaryotic translation elongation factor 1 alpha, ribosomal proteins, thioredoxin, Ras homolog, transforming growth factor beta, and vimentin were previously characterize the expression patterns in embryonic gonads as a supportive environmental niche for PGC in other species. Abdallah et al. [17] reported that the somatic form of eukaryotic translation elongation factor 1 alpha (EF-1 alpha) mRNA is virtually undetectable in male and female germ cells of the adult gonad but is very abundant in embryonic cells after the neurula stage in Xenopus laevis. Moreover, the translation pattern of the EF-1 alpha is coordinated translational regulation with ribosomal proteins in Xenopus laevis during embryogenesis [18]. It is very reasonable result of the up-regulation of antioxidant proteins, thioredoxin, in embryonic gonads because embryonic gonad is more susceptible to oxidant induced damage than adult organs. Li et al. [19] showed that in Drosophila, the receptor tyrosine kinase Torso activates both STAT and Ras during the early phase of PGC development, and co-activation of STAT and Ras is required for PGC proliferation and invasive migration. Members of the transforming growth factor (TGF) beta family are pleiotropic cytokines with key roles in tissue morphogenesis and growth and potential roles for TGF beta have been identified in gonad and secondary sex organ development, spermatogenesis and ovarian function [20]. Vimentin has been known as an immunohistological marker of Sertoli cells which shows over expression during embryonic stage [21]. In mammalian species, Ca^2+^-binding protein, calmodulin was well-known as an activator of fertilized embryo and also was closely related to regulation of interaction between germ cells and neighboring environments at embryonic stages as well as at sexual maturity [22,23].

We also conducted the alternative serial experiment for gene expression profiling using the massively parallel signature sequencing (MPSS) from PGCs and embryonic gonads (Kim *et al*., submitted). Using a FDR cut-off of 0.05, we found 4,328 and 2,681 signatures were significantly up-regulated in the PGCs and gonad sample, respectively (data not shown). Exact binomial probabilities for the situations of *n *out of *n *genes were calculated in each sample using the standard binomial formula, where the *n *is the number of genes identified using ESTs in each sample, and the probability that the differentially expressed gene of ESTs will exist in the same sample type of the MPSS is the proportion of the DES among total MPSS in each sample. Interestingly, the up-regulated signatures in the PGCs contained the two genes identified using ESTs in the PGCs. The exact binomial probability of exactly 2 out of 2 was 9.3e-4 with given DES proportion in the PGCs (3.05 %). The up-regulated signatures in the embryonic gonads also contained all of the 17 genes identified using ESTs in the embryonic gonads. The exact binomial probability of exactly 17 out of 17 was 5.01e-30 with given DES proportion in the gonads (1.89 %). Thus, although the differentially expressed genes identified with ESTs data was relatively small number, the binomial probabilities indicated that the MPSS result was very consistent with the result from the ESTs data.

## Conclusion

In this study, we could characterize expression gene profiling and identify the significant transcripts expressed in chicken primordial germ cells (PGCs) as well as embryonic gonads at 6.5 days. In the near future, serial experiments will be needed to evaluate biological function(s) and to elucidate interaction(s) in germ cells or a supportive stroma cells during the early embryo development. Germ cell is not only a unique and important cell type compared to other tissues, but also the only cell type that can penetrate from one generation to next generation. Furthermore, at the early embryonic developmental stages, the onset of proliferation and differentiation, germ cell is very tightly regulated by triggering or suppressing the essential genes. However, collection of germ cells from embryonic stages in aves is very difficulty and complicated and so there are few reports on gene transcript profiling in germ cells retrieved from the embryos to date. Thus, the results in this study would be contributed to investigating the reciprocal interaction(s) between genes during germ cell proliferation and differentiation, and accelerating novel gene mining in germ cells.

## Methods

### Retrieval of chicken embryonic gonads and MACS-separation of chicken PGCs

Experimental animals provided for this experiment were maintained at the University Animal Farm, Seoul National University, and all experimental procedures were performed at the affiliated laboratories of the university. White Leghorn (WL) embryos at 6.5 days were freed from the yolk by rinsing with calcium- and magnesium-free PBS and then embryonic gonads were retrieved by dissection of embryo abdomen with sharp tweezers under a stereomicroscope [10]. Embryonic gonads were collected from total 7,955 embryos. After collection, gonadal tissues were dissociated by gentle pipetting in 0.05% (v:v) trypsin solution supplemented with 0.53 mM EDTA. After added 10% fetal bovine serum (FBS) for inactivation of trypsin-EDTA and briefly centrifuged at 200 × g for 5 min, total gonadal cells were loaded into MACS system (Miltenyi Biotech, Germany) according to our standard protocol [12]. Breifly, chicken gonadal cells were treated with PGC-specific antibody, anti-stage specific embryo antigen (anti-SSEA)-1 antibody for chicken PGCs (mouse IgM isotype), for 20 min at the room temperature of 20–25°C. Anti-SSEA-1 antibody developed by Solter and Knowles [24] was obtained from the Developmental Studies Hybridoma Bank developed under the auspices of the NICHD and maintained by the University of Iowa, Development of Biological Science. After washing with 1 mL MACS buffer, PBS supplement with 0.5% BSA and 2 mM EDTA, the supernatant was completely removed after brief centrifugation. The cell pellet was mixed with 100 μl MACS buffer containing 20 μl of rat anti-mouse IgM microbeads for 15 min at 4°C. Treated cells were carefully washed by the addition of 500 μl buffer and subsequently loaded with MACS [12].

For counting cell number, chicken PGCs before or after MACS treatment were fixed with 1% (v:v) glutaraldehyde for 5 min and rinsed with 1× PBS twice. The anti-SSEA-1 ascites fluid diluted 1:1,000 in PBS was added and subsequent steps were carried out using DAKO universal LSAB^® ^kit, Peroxidase (DAKO, USA) according to the manufacturer's instruction.

### cDNA library construction from chicken PGCs and EST sequencing

Total RNA was extracted from MACS-separated PGCs using TRIzol reagent, and poly(A) mRNA was purified using the Promega PolyATract mRNA isolation system (Promega, WI). The cDNA libraries were synthesized using the ZAP^®^-cDNA synthesis method (Stratagene, CA). The cDNA was prepared, size-fractionated, and inserted into the Uni-ZAPXR vector using an *Xho*I linker-primer and *Eco*RI adaptor. After *in vivo *excision with *E. coli *strain SOLR, the cDNA libraries from PGCs contained inserts ranging from 0.5 to 3 kb (n = 18).

After white/blue selection, colonies were picked randomly from rectangular plates (23 × 23 cm) and transferred to 384-well plates using a Q-bot (Genetix, UK). The plasmids were purified using a Montage Plasmid Miniprep 96 kit (Millipore, MA). The sequencing reactions were performed and analyzed on ABI 3700 automated DNA sequencers (PE Applied Biosystem, CA) using the manufacturer's protocols. Macrogen (Seoul, Korea) performed all the procedures.

### ESTs processing and assembling

ESTs processing and assembling was performed as previously described (Shin et al., manuscript submitted). Briefly, in order to obtain high quality unique sequences set, relatively strict threshold criteria were applied to process the EST data. In addition, the chicken genomic sequences were used to screen out possible chimeric assembles at the final step. The chicken EST trace data were processed using the Phred [25], vector-clipped by cross match [26] and cleaned with SeqClean program at TIGR software tools [27]. The ESTs were clustered and assembled using the TIGR Gene Indices clustering tools (TGICL) [28]. To filter out chimeric assembles at the final step, the unique sequences were aligned against the chicken genomic sequences of the University of California Santa Cruz (UCSC) Genome Bioinformatics [29] using BLAT program [30]. In case of embryonic gonad ESTs, we used our previous data set (Shin et al., manuscript submitted, NCBI dbEST accession nos. CV852525-CV862818).

### Gene ontology annotation and identification of putative novel transcripts

As previously described (Shin et al., manuscript submitted), GO annotation and identification of putative novel transcripts of unique sequences was conducted on the basis of sequence similarity with the Tentative Consensus (TC) sequences of the GgGI release 10.0 (January 28, 2005) at the TIGR. The sequences with no Blast hits were represented as putative novel transcripts. GO annotation of the novel transcript was based on blast best hit against non-redundant protein database of the NCBI downloaded from NCBI blastdb download (June 29, 2005) [31]. Pearson's chi-square test was applied to test significance which GO terms were enriched in a data set but relatively depleted in the other. As described by Zhong et al. [32], a particular GO term can be viewed as a function, which maps gene G in go(G) = 0 or 1, according with the corresponding GO term. The null hypothesis of no association between gene lists and a particular GO term is translated into equal distributions of binary random variables. Only the list of genes annotated with GO terms was counted for the test. Bonferroni correction [33] was applied to correct the multiple test problems.

### Digital gene expression profiling

Significance test of gene expression profiles between a pair of the cDNA libraries was performed using Audic's [14] test. Since there was a multiple test problem, Bonferroni correction [33] was applied for the test. Number of a set of ESTs assembled into a contig was considered as the number of read of the gene. A singlet was considered as single read of the gene. Expression comparison of the gene was performed with the number of reads of the gene of two datasets. The stand alone Blast program, blastn, was used to cluster a pair of sequences between two datasets with minimum 300 bp and 95% identity.

## Authors' contributions

JH and JL conceived of the study, and participated in its design and coordination. TP carried out the molecular genetic studies and drafted the manuscript. JK and MK prepared chicken embryos and primordial germ cells, and carried out the immunoassays. DL participated in the sequence alignment and performed the statistical analysis. HK participated in its design and the statistical analysis, and drafted the manuscript. All authors read and approved the final manuscript.

## Supplementary Material

Additional File 1 Functional annotation of the novel transcripts. The list of functional annotation of the novel transcripts. Click here for file

Additional File 2 Gene Ontology annotation of the novel transcripts and Pearson's chi-square test of independent between PGCs and the novel transcripts datasets. Gene Ontology annotation of the novel transcripts (A) and Pearson's chi-square test of independent between PGCs and the novel transcripts datasets (B). Overall significance level of alpha was given by the Bonferroni correction, alpha = 0.5/m, where m was 30 since 30 GO terms were compared between gonad and the novel transcripts. Click here for file
