# Supplementary material for: Gene expression profiling of chicken primordial germ cell ESTs
Source: BMC Genomics. 2006 Aug 30;7:220. doi: 10.1186/1471-2164-7-220 (PMC1569846; doi:10.1186/1471-2164-7-220)
Supplement: Additional File 1 — Functional annotation of the novel transcripts. The list of functional annotation of the novel transcripts. [file 1471-2164-7-220-S1.doc]

**Functional annotation of the novel transcripts**

| **serial#** | **local ID** | **Best Hit description (NR database)** | **score** | **e-value** |
| --- | --- | --- | --- | --- |
| 1 | pgc_contig00014 | ref|NP_988945.1| hypothetical protein MGC75919 | 481 | e-134 |
| 2 | pgc_contig00015 | emb|CAD97644.1| hypothetical protein [Homo sapiens] | 383 | e-105 |
| 3 | pgc_contig00019 | emb|CAF93652.1| unnamed protein product [Tetraodon nigroviridis] | 69 | 2.00E-10 |
| 4 | pgc_contig00048 | gb|AAH74599.1| Unknown (protein for MGC:69468) [Xenopus tropicalis] | 153 | 1.00E-35 |
| 5 | pgc_contig00063 | sp|P18945|COX3_CHICK Cytochrome c oxidase polypeptide III | 399 | e-109 |
| 6 | pgc_contig00072 | ref|NP_990598.1| nucleophosmin; nucleolar phosphoprotein B23 | 376 | e-115 |
| 7 | pgc_contig00073 | ref|NP_990598.1| nucleophosmin; nucleolar phosphoprotein B23 | 268 | 3.00E-70 |
| 8 | pgc_contig00084 | gb|AAH46867.1| Pdip5-prov protein [Xenopus laevis] | 257 | 6.00E-67 |
| 9 | pgc_contig00091 | ref|NP_989896.1| LZ1 [Gallus gallus] | 205 | 2.00E-51 |
| 10 | pgc_contig00105 | ref|NP_990729.1| growth-related translationally controlled tumor protein | 322 | 9.00E-87 |
| 11 | pgc_contig00112 | gb|AAH70836.1| Unknown (protein for MGC:84496) [Xenopus laevis] | 144 | 4.00E-33 |
| 12 | pgc_contig00120 | ref|NP_776993.1| poly(A) binding protein, cytoplasmic 1 | 513 | e-144 |
| 13 | pgc_contig00122 | dbj|BAA74919.2| KIAA0896 protein [Homo sapiens] | 322 | 2.00E-86 |
| 14 | pgc_contig00123 | ref|NP_579932.1| SMT3 (supressor of mif two, 3) homolog 2; SMT3 | 196 | 1.00E-48 |
| 15 | pgc_contig00134 | gb|EAK88214.1| polyubiquitin with 3 Ub domains | 428 | e-118 |
| 16 | pgc_contig00135 | ref|NP_653164.1| ribose 5-phosphate isomerase A | 374 | e-102 |
| 17 | pgc_singlet00014 | ref|NP_570106.1| Gasz [Rattus norvegicus] | 215 | 2.00E-54 |
| 18 | pgc_singlet00016 | gb|AAH01336.1| Similar to nectin 3; DKFZP566B0846 protein | 275 | 2.00E-72 |
| 19 | pgc_singlet00017 | dbj|BAC05047.1| unnamed protein product [Homo sapiens] | 267 | 4.00E-70 |
| 20 | pgc_singlet00029 | ref|XP_345233.1| similar to CDC-like kinase 2 [Rattus norvegicus] | 82 | 3.00E-14 |
| 21 | pgc_singlet00038 | ref|NP_872578.1| mitochondrial ribosomal protein S9; 28S ribosomal protein S9 | 112 | 1.00E-23 |
| 22 | pgc_singlet00039 | ref|NP_149078.1| hypothetical protein FLJ14511 isoform 1 | 238 | 2.00E-61 |
| 23 | pgc_singlet00041 | dbj|BAB71102.1| unnamed protein product [Homo sapiens] | 617 | e-175 |
| 24 | pgc_singlet00044 | gb|AAD08679.1| NNX3 [Homo sapiens] | 267 | 4.00E-70 |
| 25 | pgc_singlet00050 | ref|NP_006539.2| IGF-II mRNA-binding protein 2 [Homo sapiens] | 349 | 7.00E-95 |
| 26 | pgc_singlet00055 | dbj|BAC41403.1| mKIAA0240 protein [Mus musculus] | 490 | e-137 |
| 27 | pgc_singlet00062 | gb|AAK92205.1| histone deacetylase 10 isoform alpha [Homo sapiens] | 72 | 2.00E-11 |
| 28 | pgc_singlet00064 | ref|NP_990846.1| actin-capping protein (CapZ 36/32) alpha subunit | 550 | e-155 |
| 29 | pgc_singlet00069 | dbj|BAA20772.2| KIAA0313 [Homo sapiens] | 56 | 2.00E-06 |
| 30 | pgc_singlet00070 | ref|NP_775836.1| hypothetical protein LOC222967 [Homo sapiens] | 421 | e-116 |
| 31 | pgc_singlet00071 | emb|CAF92178.1| unnamed protein product [Tetraodon nigroviridis] | 75 | 3.00E-12 |
| 32 | pgc_singlet00072 | gb|AAH62515.1| LOC394691 protein [Xenopus tropicalis] | 186 | 7.00E-46 |
| 33 | pgc_singlet00073 | ref|NP_031895.1| DnaJ (Hsp40) homolog, subfamily C, member 1;DnaJ-like protein 1 | 105 | 4.00E-29 |
| 34 | pgc_singlet00076 | sp|P48440|OST4_CHICK Dolichyl-diphosphooligosaccharide | 364 | e-130 |
| 35 | pgc_singlet00088 | ref|XP_356960.1| expressed sequence AV101767 [Mus musculus] | 292 | 1.00E-77 |
| 36 | pgc_singlet00103 | gb|AAC60281.1| unknown [Gallus gallus] | 62 | 1.00E-23 |
| 37 | pgc_singlet00118 | dbj|BAC05047.1| unnamed protein product [Homo sapiens] | 421 | e-116 |
| 38 | pgc_singlet00142 | emb|CAB62528.1| hypothetical protein [Homo sapiens] | 154 | 5.00E-36 |
| 39 | pgc_singlet00151 | gb|AAF65544.1| 170 kDa glucose regulated protein GRP170 precursor | 177 | 2.00E-68 |
| 40 | pgc_singlet00154 | gb|AAG45219.1| retinoblastoma tumor suppressor [Gallus gallus] | 75 | 2.00E-12 |
| 41 | pgc_singlet00155 | ref|NP_989501.1| proliferating cell nuclear antigen | 384 | e-105 |
| 42 | pgc_singlet00166 | gb|AAH44001.1| MGC53359 protein [Xenopus laevis] | 580 | e-175 |
| 43 | pgc_singlet00171 | ref|XP_217233.2| similar to 5-3 exonuclease [Rattus norvegicus] | 259 | 1.00E-67 |
| 44 | pgc_singlet00172 | gb|AAN86071.1| heat-sensitive lambda citS857 repressor protein/P16 fusion protein | 451 | e-125 |
| 45 | pgc_singlet00174 | gb|AAH55761.1| 6720435I21Rik protein [Mus musculus] | 97 | 2.00E-20 |
| 46 | pgc_singlet00175 | ref|XP_342455.1| similar to Ierepo4-pending protein | 317 | e-119 |
| 47 | pgc_singlet00181 | gb|AAA99015.1| cyclin-dependent kinase | 123 | 7.00E-27 |
| 48 | pgc_singlet00184 | ref|NP_766253.1| RIKEN cDNA D530020C15 [Mus musculus] | 103 | 5.00E-39 |
| 49 | pgc_singlet00186 | gb|AAR28760.1| DNA-dependent protein kinase catalytic subunit | 72 | 4.00E-21 |
| 50 | pgc_singlet00204 | sp|P25391|LMA1_HUMAN Laminin alpha-1 chain precursor | 391 | e-107 |
| 51 | pgc_singlet00211 | ref|NP_113602.2| serine/threonine kinase 31 isoform a | 360 | 3.00E-98 |
| 52 | pgc_singlet00214 | ref|NP_757386.2| splicing factor 4 isoform a; RNA-binding protein | 273 | 5.00E-72 |
| 53 | pgc_singlet00217 | gb|AAR28760.1| DNA-dependent protein kinase catalytic subunit | 89 | 2.00E-16 |
| 54 | pgc_singlet00219 | sp|P47859|K6PP_RABIT 6-phosphofructokinase, type C | 100 | 9.00E-20 |
| 55 | pgc_singlet00225 | ref|NP_113980.1| sperm antigen 4 [Rattus norvegicus] | 169 | 1.00E-40 |
| 56 | pgc_singlet00233 | ref|NP_004232.1| jumonji domain containing 1C; thyroid receptor interacting protein 8 | 505 | e-142 |
| 57 | pgc_singlet00247 | emb|CAA46671.1| Hox-7 [Gallus gallus] | 216 | 1.00E-54 |
| 58 | pgc_singlet00251 | ref|NP_078937.2| hypothetical protein FLJ12436 [Homo sapiens] | 169 | 2.00E-40 |
| 59 | pgc_singlet00252 | dbj|BAC85852.1| unnamed protein product [Homo sapiens] | 409 | e-113 |
| 60 | pgc_singlet00253 | gb|AAC60281.1| unknown [Gallus gallus] | 114 | 7.00E-28 |
| 61 | pgc_singlet00255 | emb|CAG02887.1| unnamed protein product [Tetraodon nigroviridis] | 157 | 4.00E-37 |
| 62 | pgc_singlet00261 | ref|NP_112384.1| suppression of tumorigenicity 13 (colon carcinoma) | 102 | 1.00E-20 |
| 63 | pgc_singlet00264 | dbj|BAC86991.1| unnamed protein product [Homo sapiens] | 234 | 3.00E-60 |
| 64 | pgc_singlet00270 | emb|CAG02003.1| unnamed protein product [Tetraodon nigroviridis] | 151 | 3.00E-35 |
| 65 | pgc_singlet00272 | dbj|BAC40418.1| unnamed protein product [Mus musculus] | 55 | 3.00E-06 |
| 66 | pgc_singlet00274 | ref|XP_376722.1| similar to RIKEN cDNA 1700025J14 [Homo sapiens] | 204 | 4.00E-51 |
| 67 | pgc_singlet00285 | pir||JC1129 nonhistone chromosomal protein HMG-2 - chicken | 220 | 6.00E-56 |
| 68 | pgc_singlet00289 | gb|AAR28760.1| DNA-dependent protein kinase catalytic subunit | 95 | 4.00E-18 |
| 69 | pgc_singlet00297 | gb|AAK83376.1| CRSP70-like protein [Xenopus laevis] | 248 | 2.00E-64 |
| 70 | pgc_singlet00299 | ref|NP_808908.1| protein phosphatase 1B isoform 3 | 215 | 2.00E-54 |
| 71 | pgc_singlet00302 | sp|Q91V51|TTL1_MOUSE Tubulin tyrosine ligase-like protein 1 | 344 | 2.00E-93 |
| 72 | pgc_singlet00306 | gb|AAH24612.2| RIKEN cDNA 5730434I03 gene [Mus musculus] | 182 | 1.00E-61 |
| 73 | pgc_singlet00315 | emb|CAA61536.1| anti-mullerian hormone [Gallus gallus] | 158 | 2.00E-37 |
| 74 | pgc_singlet00320 | dbj|BAB59062.1| Pex1pL664P [Homo sapiens] | 285 | 1.00E-80 |
| 75 | pgc_singlet00324 | gb|AAH68662.1| MGC81046 protein [Xenopus laevis] | 105 | 2.00E-21 |
| 76 | pgc_singlet00348 | dbj|BAB15608.1| unnamed protein product [Homo sapiens] | 249 | 1.00E-64 |
| 77 | pgc_singlet00349 | dbj|BAC27440.1| unnamed protein product [Mus musculus] | 161 | 3.00E-38 |
| 78 | pgc_singlet00350 | ref|NP_988845.1| SAR1a protein [Xenopus tropicalis] | 400 | e-110 |
| 79 | pgc_singlet00351 | ref|NP_003615.1| RAN binding protein 3 isoform RANBP3-a | 306 | 7.00E-82 |
| 80 | pgc_singlet00355 | ref|NP_689543.1| chromosome 14 open reading frame 31 | 412 | e-113 |
| 81 | pgc_singlet00360 | ref|NP_000652.2| ribosomal protein L9; 60S ribosomal protein L9 | 347 | 3.00E-94 |
| 82 | pgc_singlet00362 | dbj|BAA13209.2| similar to Yeast translation activator GCN1 | 496 | e-139 |
| 83 | pgc_singlet00375 | emb|CAC27349.1| bA287B20.1.1 (KIAA1272 similar to rat tulip protein 1 and 2 | 450 | e-125 |
| 84 | pgc_singlet00378 | ref|NP_000851.2| hydroxyprostaglandin dehydrogenase 15-(NAD) | 364 | 2.00E-99 |
| 85 | pgc_singlet00382 | gb|AAT06732.1| L2 [Homo sapiens] | 170 | 7.00E-41 |
| 86 | pgc_singlet00385 | gb|AAF08220.1| ALG-2 interacting protein 1 [Homo sapiens] | 345 | 2.00E-93 |
| 87 | pgc_singlet00389 | ref|XP_235625.2| similar to hypothetical protein FLJ31295 | 65 | 3.00E-09 |
| 88 | pgc_singlet00392 | ref|XP_217476.1| hypothetical protein XP_217476 [Rattus norvegicus] | 76 | 2.00E-12 |
| 89 | pgc_singlet00395 | dbj|BAA34453.2| KIAA0733 protein [Homo sapiens] | 71 | 5.00E-11 |
| 90 | pgc_singlet00403 | dbj|BAA13399.2| KIAA0269 [Homo sapiens] | 505 | e-141 |
| 91 | pgc_singlet00406 | dbj|BAA34432.2| KIAA0712 protein [Homo sapiens] | 135 | 2.00E-30 |
| 92 | pgc_singlet00408 | emb|CAG12949.1| unnamed protein product [Tetraodon nigroviridis] | 238 | 3.00E-61 |
| 93 | pgc_singlet00409 | ref|NP_079522.1| transmembrane protein 22 [Homo sapiens] | 83 | 8.00E-22 |
| 94 | pgc_singlet00424 | ref|NP_989756.1| cysteine-rich motorneuron 1 [Gallus gallus] | 375 | e-102 |
| 95 | pgc_singlet00425 | ref|NP_057199.1| ankyrin repeat and SOCS box-containing protein | 157 | 6.00E-37 |
| 96 | pgc_singlet00429 | ref|NP_079521.1| pre-B-cell leukemia transcription factor 4 | 411 | e-113 |
| 97 | pgc_singlet00436 | ref|XP_213602.2| similar to hypothetical protein FLJ20522 | 125 | 2.00E-27 |
| 98 | pgc_singlet00437 | ref|NP_733468.1| AF15q14 protein [Homo sapiens] | 254 | 9.00E-67 |
| 99 | pgc_singlet00442 | dbj|BAC98182.1| mKIAA1486 protein [Mus musculus] | 301 | 2.00E-80 |
| 100 | pgc_singlet00458 | emb|CAA53980.2| nectin 1 [Homo sapiens] | 381 | e-104 |
| 101 | pgc_singlet00459 | ref|NP_877435.1| hypothetical protein FLJ38736 [Homo sapiens] | 278 | 2.00E-73 |
| 102 | pgc_singlet00483 | ref|NP_766252.1| tousled-like kinase 1 [Mus musculus] | 464 | e-129 |
| 103 | pgc_singlet00488 | emb|CAF99207.1| unnamed protein product [Tetraodon nigroviridis] | 85 | 3.00E-15 |
| 104 | pgc_singlet00492 | ref|NP_689718.1| chromosome 21 open reading frame 13; hypothetical 76.5 kD protein | 192 | 1.00E-47 |
| 105 | pgc_singlet00511 | ref|NP_009191.1| lysophospholipase II; acyl-protein thioesterase | 235 | 1.00E-84 |
| 106 | pgc_singlet00518 | dbj|BAB69710.1| hypothetical protein [Macaca fascicularis] | 102 | 2.00E-20 |
| 107 | pgc_singlet00519 | gb|AAC60281.1| unknown [Gallus gallus] | 105 | 1.00E-29 |
| 108 | pgc_singlet00527 | ref|NP_989818.1| double-stranded RNA-activated protein kinase | 158 | 2.00E-37 |
| 109 | pgc_singlet00530 | ref|XP_355454.1| similar to DKFZP434D146 protein [Mus musculus] | 223 | 9.00E-57 |
| 110 | pgc_singlet00535 | ref|NP_073583.2| hypothetical protein FLJ22390 [Homo sapiens] | 211 | 4.00E-53 |
| 111 | pgc_singlet00537 | ref|NP_932122.1| RIKEN cDNA 4833418A01 [Mus musculus] | 474 | e-132 |
| 112 | pgc_singlet00538 | ref|NP_989612.1| SRY (sex determining region Y)-box 9 (campomelic dysplasia) | 432 | e-120 |
| 113 | pgc_singlet00541 | ref|NP_940916.2| NHL repeat containing 2; Similar to RIKEN cDNA 1200003G01 gene | 133 | 1.00E-29 |
| 114 | pgc_singlet00547 | ref|XP_136170.3| similar to zinc finger protein 91 (HPF7, HTF10) | 321 | 2.00E-86 |
| 115 | pgc_singlet00550 | dbj|BAC56558.1| similar to ribosomal protein L3 [Bos taurus] | 69 | 3.00E-10 |
| 116 | pgc_singlet00585 | gb|AAG12466.1| Kruppel-type zinc finger protein KROX-25 | 105 | 2.00E-21 |
| 117 | pgc_singlet00590 | gb|AAQ88552.1| olfactomedin-like [Homo sapiens] | 414 | e-119 |
| 118 | pgc_singlet00598 | gb|AAH60656.1| RNA polymerase I polypeptide B [Mus musculus] | 376 | e-103 |
| 119 | pgc_singlet00602 | ref|NP_990371.1| DGCR6 homolog [Gallus gallus] | 358 | 2.00E-97 |
| 120 | pgc_singlet00603 | gb|AAH17247.1| FUBP1 protein [Homo sapiens] | 91 | 6.00E-17 |
| 121 | pgc_singlet00606 | sp|Q9DE46|DPOA_XENLA DNA polymerase alpha catalytic subunit | 446 | e-124 |
| 122 | pgc_singlet00611 | ref|NP_787061.1| transcription factor ELYS [Homo sapiens] | 276 | e-112 |
| 123 | pgc_singlet00612 | gb|AAC60281.1| unknown [Gallus gallus] | 89 | 3.00E-16 |
| 124 | pgc_singlet00616 | ref|NP_990699.1| elongation factor 2 [Gallus gallus] | 642 | 0 |
| 125 | pgc_singlet00618 | ref|NP_776415.1| S-adenosylmethionine decarboxylase 1 | 521 | e-154 |
| 126 | pgc_singlet00631 | emb|CAA61536.1| anti-mullerian hormone [Gallus gallus] | 143 | 8.00E-33 |
| 127 | pgc_singlet00644 | ref|NP_055847.1| androgen-induced prostate proliferative shutoff associated protein | 183 | 6.00E-45 |
| 128 | pgc_singlet00648 | ref|XP_397265.1| similar to elicitor-like mating protein M81 (2A987) | 73 | 2.00E-11 |
| 129 | pgc_singlet00654 | ref|XP_287555.2| similar to KIAA0960 protein [Mus musculus] | 491 | e-137 |
| 130 | pgc_singlet00665 | ref|NP_570106.1| Gasz [Rattus norvegicus] | 371 | e-101 |
| 131 | pgc_singlet00667 | ref|NP_851851.1| Rho GTPase activating protein 8 isoform 1 | 338 | e-106 |
| 132 | pgc_singlet00668 | ref|NP_035287.1| proteoglycan, secretory granule [Mus musculus] | 57 | 5.00E-07 |
| 133 | pgc_singlet00671 | ref|XP_341756.1| similar to Wilms tumour 1-associating protein isoform 2 | 264 | 3.00E-69 |
| 134 | pgc_singlet00690 | pir||B36203 iron-responsive element-binding protein (clone 10.1) | 96 | 2.00E-18 |
| 135 | pgc_singlet00693 | ref|XP_341375.1| similar to putative MAPK activating protein | 90 | 1.00E-16 |
| 136 | pgc_singlet00697 | ref|NP_996745.1| vav 3 oncogene [Gallus gallus] | 317 | 5.00E-85 |
| 137 | pgc_singlet00701 | pir||RDHUP dihydropteridine reductase (EC 1.6.99.7) [validated] | 193 | 6.00E-48 |
| 138 | pgc_singlet00706 | gb|AAA51779.1| arylsulfatase B precursor | 342 | e-102 |
| 139 | pgc_singlet00709 | pir||S23298 collagen alpha 1(VIII) chain - chicken | 94 | 5.00E-18 |
| 140 | pgc_singlet00712 | ref|NP_570955.1| oxidation resistance 1; nucleolar protein C7 | 97 | 9.00E-33 |
| 141 | pgc_singlet00719 | ref|NP_006545.1| metaxin 2 [Homo sapiens] | 474 | e-132 |
| 142 | pgc_singlet00728 | gb|AAH60656.1| RNA polymerase I polypeptide B [Mus musculus] | 423 | e-128 |
| 143 | pgc_singlet00729 | ref|NP_989603.1| ribosomal protein L39 [Gallus gallus] | 105 | 2.00E-21 |
| 144 | pgc_singlet00731 | ref|NP_000040.1| aspartoacylase; aminoacylase 2 [Homo sapiens] | 410 | e-113 |
| 145 | pgc_singlet00735 | ref|XP_344483.1| similar to PHD finger protein 7 isoform 1 | 140 | 6.00E-32 |
| 146 | pgc_singlet00737 | ref|NP_064548.1| semaphorin sem2 [Homo sapiens] | 89 | 1.00E-36 |
| 147 | pgc_singlet00744 | pir||T42673 hypothetical protein DKFZp434L0217.1 - human (fragment) | 123 | 1.00E-26 |
| 148 | pgc_singlet00748 | ref|NP_036213.1| elongation factor, RNA polymerase II, 2 | 432 | e-126 |
| 149 | pgc_singlet00751 | gb|AAH23767.1| Eif3s9 protein [Mus musculus] | 350 | 5.00E-95 |
| 150 | pgc_singlet00752 | ref|NP_689589.1| UBX domain containing 3 [Homo sapiens] | 108 | 3.00E-22 |
| 151 | pgc_singlet00754 | ref|NP_036276.1| dynein, axonemal, intermediate polypeptide 1 | 290 | 5.00E-77 |
| 152 | pgc_singlet00756 | pdb|1U9A|A Chain A, Human Ubiquitin-Conjugating Enzyme Ubc9 | 340 | 6.00E-92 |
| 153 | pgc_singlet00764 | ref|NP_033904.2| budding uninhibited by benzimidazoles 3 homolog | 111 | 3.00E-23 |
| 154 | pgc_singlet00769 | dbj|BAB93526.1| ribosomal protein S2 [Homo sapiens] | 68 | 6.00E-14 |
| 155 | pgc_singlet00777 | ref|NP_005527.1| inositol(myo)-1(or 4)-monophosphatase 1 | 408 | e-112 |
| 156 | pgc_singlet00784 | ref|NP_001673.2| plasma membrane calcium ATPase 1 isoform 1b | 468 | e-139 |
| 157 | pgc_singlet00785 | ref|XP_233441.2| similar to KIAA0677 gene product | 98 | 4.00E-19 |
| 158 | pgc_singlet00789 | ref|XP_193940.1| RIKEN cDNA 2410089B13 [Mus musculus] | 215 | 2.00E-54 |
| 159 | pgc_singlet00800 | emb|CAD97846.1| hypothetical protein [Homo sapiens] | 530 | e-149 |
| 160 | pgc_singlet00801 | emb|CAG10177.1| unnamed protein product [Tetraodon nigroviridis] | 168 | 3.00E-40 |
| 161 | pgc_singlet00808 | gb|AAH74376.1| Unknown (protein for MGC:84293) [Xenopus laevis] | 99 | 2.00E-19 |
| 162 | pgc_singlet00813 | ref|XP_374625.1| similar to hypothetical protein [Homo sapiens] | 268 | 2.00E-72 |
| 163 | pgc_singlet00818 | ref|NP_989530.1| survival motor neuron [Gallus gallus] | 173 | 9.00E-42 |
| 164 | pgc_singlet00820 | gb|AAH43464.1| Similar to multiple PDZ domain protein | 319 | 1.00E-85 |
| 165 | pgc_singlet00826 | gb|AAH43827.1| Slc25a20-prov protein [Xenopus laevis] | 387 | e-106 |
| 166 | pgc_singlet00828 | ref|NP_612434.1| C10 protein; C10 gene [Homo sapiens] | 186 | 6.00E-46 |
| 167 | pgc_singlet00830 | gb|AAH61015.1| Unknown (protein for MGC:74120) [Mus musculus] | 275 | 2.00E-72 |
| 168 | pgc_singlet00839 | pir||T08708 hypothetical protein DKFZp564D116.1 - human (fragment) | 62 | 3.00E-08 |
| 169 | pgc_singlet00840 | ref|NP_989896.1| LZ1 [Gallus gallus] | 258 | 2.00E-67 |
| 170 | pgc_singlet00846 | dbj|BAA92661.1| KIAA1423 protein [Homo sapiens] | 159 | 2.00E-37 |
| 171 | pgc_singlet00854 | gb|AAH59990.1| MGC68696 protein [Xenopus laevis] | 345 | e-109 |
| 172 | pgc_singlet00871 | ref|XP_294139.2| similar to expressed sequence AA409316 | 178 | 3.00E-43 |
| 173 | pgc_singlet00873 | dbj|BAA88337.1| ORF2 [Platemys spixii] | 237 | 1.00E-73 |
| 174 | pgc_singlet00875 | gb|AAH73532.1| Unknown (protein for MGC:82793) [Xenopus laevis] | 390 | e-107 |
| 175 | pgc_singlet00876 | ref|YP_031678.1| Gag-env fusion protein [Avian endogenous retrovirus EAV-HP | 222 | 2.00E-56 |
| 176 | pgc_singlet00883 | gb|AAB06721.1| aldehyde dehydrogenase E3' | 465 | e-130 |
| 177 | pgc_singlet00885 | ref|XP_341606.1| similar to KIAA1311 protein [Rattus norvegicus] | 227 | 5.00E-58 |
| 178 | pgc_singlet00887 | sp|Q8WZA2|EPC2_HUMAN cAMP-regulated guanine nucleotide exchange factor II | 558 | e-157 |
| 179 | pgc_singlet00891 | gb|AAH70405.1| Unknown (protein for MGC:99440) [Mus musculus] | 59 | 2.00E-07 |
| 180 | pgc_singlet00896 | dbj|BAC98146.1| mKIAA1341 protein [Mus musculus] | 214 | 5.00E-84 |
| 181 | pgc_singlet00899 | ref|XP_373440.2| chondroitin sulfate synthase 3 [Homo sapiens] | 488 | e-150 |
| 182 | pgc_singlet00900 | ref|NP_777619.1| hypothetical protein LOC136306 [Homo sapiens] | 191 | 3.00E-47 |
| 183 | pgc_singlet00902 | gb|AAH64930.1| ATP-binding cassette, sub-family B, member 10 | 407 | e-127 |
| 184 | pgc_singlet00908 | ref|NP_665710.1| paired-like homeodomain trancription factor Drg11 | 347 | 4.00E-94 |
| 185 | pgc_singlet00910 | gb|AAH32966.1| BC031353 protein [Mus musculus] | 80 | 3.00E-14 |
| 186 | pgc_singlet00930 | dbj|BAB13396.1| KIAA1570 protein [Homo sapiens] | 234 | 4.00E-60 |
| 187 | pgc_singlet00932 | ref|NP_694949.1| hypothetical protein MGC13034 [Homo sapiens] | 233 | 5.00E-60 |
| 188 | pgc_singlet00942 | ref|XP_342398.1| similar to hypothetical protein | 544 | e-153 |
| 189 | pgc_singlet00945 | sp|Q62448|I4G2_MOUSE Eukaryotic translation initiation factor 4 gamma 2 | 345 | 5.00E-96 |
| 190 | pgc_singlet00950 | pir||S23104 choline kinase - human | 558 | e-157 |
| 191 | pgc_singlet00951 | ref|NP_061060.3| chondroitin beta1,4 N-acetylgalactosaminyltransferase 2 | 417 | e-115 |
| 192 | pgc_singlet00959 | gb|AAH22050.1| Unknown (protein for IMAGE:4331969) [Homo sapiens] | 555 | e-157 |
| 193 | pgc_singlet00961 | ref|XP_343104.1| similar to 2900070E19Rik protein | 81 | 5.00E-14 |
| 194 | pgc_singlet00962 | gb|AAH73659.1| Unknown (protein for MGC:83004) [Xenopus laevis] | 293 | 5.00E-78 |
| 195 | pgc_singlet00969 | emb|CAE75663.1| amyloid-beta-like protein B precursor | 217 | 4.00E-55 |
| 196 | pgc_singlet00970 | gb|AAF73953.2| acetyltransferase Tubedown-1 [Mus musculus] | 101 | 4.00E-20 |
| 197 | pgc_singlet00973 | ref|NP_061887.2| RNA polymerase I polypeptide B; DNA-directed RNA polymerase I | 435 | e-142 |
| 198 | pgc_singlet00982 | ref|NP_112275.1| liver multidrug resistance-associated protein 6 | 52 | 7.00E-09 |
| 199 | pgc_singlet00983 | pir||T17229 hypothetical protein DKFZp434D156.1 - human | 176 | 1.00E-42 |
| 200 | pgc_singlet00994 | ref|NP_991368.1| lysosomal-associated protein transmembrane 4 alpha | 364 | 3.00E-99 |
| 201 | pgc_singlet01010 | dbj|BAA04878.2| KIAA0029 [Homo sapiens] | 173 | 6.00E-42 |
| 202 | pgc_singlet01013 | ref|NP_996772.1| DIP2-like protein isoform b; disco-interacting protein 2 | 71 | 5.00E-11 |
| 203 | pgc_singlet01035 | ref|NP_034969.1| mature T-cell proliferation 1 [Mus musculus] | 67 | 1.00E-09 |
| 204 | pgc_singlet01036 | ref|NP_705242.1| hypothetical protein [Plasmodium falciparum 3D7] | 56 | 2.00E-06 |
| 205 | pgc_singlet01042 | gb|AAG45219.1| retinoblastoma tumor suppressor [Gallus gallus] | 51 | 9.00E-06 |
| 206 | pgc_singlet01052 | dbj|BAA88337.1| ORF2 [Platemys spixii] | 117 | 2.00E-42 |
| 207 | pgc_singlet01055 | dbj|BAC11215.1| unnamed protein product [Homo sapiens] | 259 | 8.00E-68 |
| 208 | pgc_singlet01060 | dbj|BAA84924.1| ABP130 [Homo sapiens] | 174 | 5.00E-42 |
| 209 | pgc_singlet01061 | ref|NP_612378.1| hypothetical protein BC007436 [Homo sapiens] | 243 | 7.00E-63 |
| 210 | pgc_singlet01065 | ref|NP_004807.2| chromosome 9 open reading frame 61 | 304 | 2.00E-81 |
| 211 | pgc_singlet01079 | gb|AAH36023.1| Polo-like kinase 4 [Homo sapiens] | 223 | 1.00E-56 |
| 212 | pgc_singlet01085 | ref|XP_213980.1| similar to ATPase, H+ transporting, V1 subunit G, isoform 3 | 99 | 3.00E-19 |
| 213 | pgc_singlet01092 | ref|NP_004676.2| myotubularin related protein 6 [Homo sapiens] | 365 | e-100 |
| 214 | pgc_singlet01097 | ref|NP_057930.1| IQ motif containing GTPase activating protein 1 | 128 | 2.00E-28 |
| 215 | pgc_singlet01104 | ref|NP_033151.1| spinocerebellar ataxia 2 homolog; ataxin 2 | 379 | e-104 |
| 216 | pgc_singlet01108 | ref|NP_796085.2| RIKEN cDNA E230015L20 gene [Mus musculus] | 275 | 1.00E-72 |
| 217 | pgc_singlet01110 | emb|CAG10582.1| unnamed protein product [Tetraodon nigroviridis] | 252 | 2.00E-65 |
| 218 | pgc_singlet01111 | ref|XP_344483.1| similar to PHD finger protein 7 isoform 1 | 102 | 2.00E-20 |
| 219 | pgc_singlet01112 | dbj|BAC55020.1| N-acetylglucosaminyltrasnferase IVb [Mus musculus] | 392 | e-108 |
| 220 | pgc_singlet01114 | ref|NP_990799.1| p80/85 [Gallus gallus] | 81 | 6.00E-14 |
| 221 | pgc_singlet01135 | ref|XP_289903.2| similar to KIAA1285 protein [Mus musculus] | 167 | 3.00E-40 |
| 222 | pgc_singlet01147 | ref|XP_228159.2| similar to DKFZP586B0923 protein | 358 | 2.00E-97 |
| 223 | pgc_singlet01152 | ref|NP_612356.1| zinc finger protein 551 [Homo sapiens] | 392 | e-107 |
| 224 | pgc_singlet01159 | ref|NP_990035.1| RhoA GTPase [Gallus gallus] | 390 | e-107 |
| 225 | pgc_singlet01175 | emb|CAB87612.1| dJ309K20.5 (continues in bA353C18 (AL357374)) | 150 | 6.00E-35 |
| 226 | pgc_singlet01179 | gb|AAH08579.2| NMT1 protein [Homo sapiens] | 208 | 2.00E-52 |
| 227 | pgc_singlet01185 | ref|NP_005871.1| DnaJ subfamily A member 2; cell cycle progression 3 protein | 411 | e-113 |
| 228 | pgc_singlet01204 | ref|NP_689542.2| LRR-repeat protein 1 isoform 1 | 295 | 2.00E-78 |
| 229 | pgc_singlet01207 | dbj|BAA92079.1| unnamed protein product [Homo sapiens] | 132 | 1.00E-29 |
| 230 | pgc_singlet01211 | ref|NP_002363.1| mannosidase, alpha, class 2A, member 1 | 444 | e-123 |
| 231 | pgc_singlet01226 | ref|XP_342690.1| similar to RIKEN cDNA 1200009O22; EST AI316813 | 158 | 3.00E-37 |
| 232 | pgc_singlet01232 | ref|NP_114437.1| elastin microfibril interfacer 2 | 179 | 1.00E-43 |
| 233 | pgc_singlet01246 | ref|XP_343165.1| similar to DAZ associated protein 1 isoform b | 87 | 7.00E-16 |
| 234 | pgc_singlet01250 | gb|AAP97322.1| unknown [Homo sapiens] | 436 | e-121 |
| 235 | pgc_singlet01261 | ref|XP_355951.1| TRAF-binding protein [Mus musculus] | 538 | e-152 |
| 236 | pgc_singlet01272 | ref|NP_705810.2| chemokine-like factor super family 4 | 326 | 8.00E-88 |
| 237 | pgc_singlet01273 | dbj|BAA74905.2| KIAA0882 protein [Homo sapiens] | 167 | 6.00E-40 |
| 238 | pgc_singlet01274 | ref|NP_060516.2| angiogenic factor VG5Q; vasculogenesis gene on 5q | 80 | 1.00E-13 |
| 239 | pgc_singlet01278 | sp|P26429|SL51_PIG Sodium/glucose cotransporter 1 (Na(+)/glucose cotransporter 1) | 79 | 3.00E-13 |
| 240 | pgc_singlet01280 | ref|NP_062174.1| complement receptor related protein | 127 | 3.00E-32 |
| 241 | pgc_singlet01281 | sp|Q95KC8|TIP_MACFA T-cell immunomodulatory protein (TIP protein) | 417 | e-115 |
| 242 | pgc_singlet01282 | ref|NP_989544.1| cyclin D2 [Gallus gallus] | 142 | 2.00E-32 |
| 243 | pgc_singlet01286 | gb|AAG45219.1| retinoblastoma tumor suppressor [Gallus gallus] | 61 | 3.00E-15 |
| 244 | pgc_singlet01288 | gb|AAF66443.1| unknown [Homo sapiens] | 85 | 4.00E-15 |
| 245 | pgc_singlet01291 | sp|Q04791|SASB_ANAPL Fatty acyl-CoA hydrolase precursor, medium chain | 111 | 4.00E-23 |
| 246 | pgc_singlet01296 | gb|AAC60281.1| unknown [Gallus gallus] | 74 | 3.00E-17 |
| 247 | pgc_singlet01303 | ref|NP_038880.1| ATP-binding cassette, sub-family F (GCN20), member 3 | 352 | 1.00E-95 |
| 248 | pgc_singlet01304 | ref|NP_065178.1| actin-related protein 3-beta; actin-related protein Arp11 | 555 | e-166 |
| 249 | pgc_singlet01309 | ref|NP_443730.1| guanine nucleotide binding protein beta-subunit-like polypeptide | 128 | 3.00E-28 |
| 250 | pgc_singlet01315 | ref|NP_989660.1| GCN5 general control of amino-acid synthesis 5-like 2 | 398 | e-109 |
| 251 | pgc_singlet01328 | dbj|BAC27743.1| unnamed protein product [Mus musculus] | 459 | e-128 |
| 252 | pgc_singlet01331 | gb|AAH72730.1| Unknown (protein for MGC:79074) [Xenopus laevis] | 484 | e-135 |
| 253 | pgc_singlet01332 | emb|CAB09782.1| dJ179M20.1 (Adenosine deaminase) [Homo sapiens] | 56 | 2.00E-06 |
| 254 | pgc_singlet01334 | ref|NP_951062.1| DEAD box polypeptide 17 isoform 1 [Mus musculus] | 167 | 4.00E-40 |
| 255 | pgc_singlet01336 | gb|AAC39582.1| unknown [Homo sapiens] | 112 | 2.00E-23 |
| 256 | pgc_singlet01338 | gb|AAH74231.1| Unknown (protein for MGC:83430) [Xenopus laevis] | 116 | 1.00E-24 |
| 257 | pgc_singlet01346 | gb|AAF36723.1| TRIAD3 [Homo sapiens] | 182 | 2.00E-44 |
| 258 | pgc_singlet01348 | gb|AAH36372.1| Zinc finger protein 198 [Homo sapiens] | 374 | e-102 |
| 259 | pgc_singlet01351 | ref|NP_999182.1| aminopeptidase A [Sus scrofa] | 359 | 6.00E-98 |
| 260 | pgc_singlet01353 | sp|P13696|PEBP_BOVIN Phosphatidylethanolamine-binding protein (PEBP) | 335 | 1.00E-90 |
| 261 | pgc_singlet01357 | ref|NP_694765.1| hypothetical protein MGC38812 [Mus musculus] | 151 | 4.00E-35 |
| 262 | pgc_singlet01361 | ref|NP_766073.2| RIKEN cDNA D130067I03 [Mus musculus] | 434 | e-120 |
| 263 | pgc_singlet01366 | ref|NP_002938.1| replication protein A3, 14kDa; replication protein A3 | 98 | 3.00E-19 |
| 264 | pgc_singlet01367 | gb|AAH74298.1| Unknown (protein for MGC:84100) [Xenopus laevis] | 336 | 8.00E-91 |
| 265 | pgc_singlet01369 | ref|NP_990863.1| initiation factor 5A [Gallus gallus] | 244 | 3.00E-63 |
| 266 | pgc_singlet01381 | ref|XP_216525.2| hypothetical protein XP_216525 [Rattus norvegicus] | 145 | 2.00E-51 |
| 267 | pgc_singlet01387 | ref|NP_064508.2| endomembrane protein emp70 precursor isolog | 67 | 5.00E-19 |
| 268 | pgc_singlet01391 | dbj|BAA07527.1| KIAA0080 [Homo sapiens] | 206 | 7.00E-52 |
| 269 | pgc_singlet01395 | ref|NP_689753.2| sec1 family domain containing 2 | 295 | 2.00E-78 |
| 270 | pgc_singlet01402 | dbj|BAC11842.1| HIRA [Gallus gallus] | 415 | e-114 |
| 271 | pgc_singlet01403 | ref|NP_190891.1| cell division cycle protein 48, putative / CDC48 | 69 | 2.00E-10 |
| 272 | pgc_singlet01407 | gb|AAH08369.1| STAU2 protein [Homo sapiens] | 141 | 3.00E-32 |
| 273 | pgc_singlet01408 | gb|AAN76189.1| BLOCK24 variant [Homo sapiens] | 336 | 6.00E-91 |
| 274 | pgc_singlet01409 | ref|XP_110787.2| UPF3 regulator of nonsense transcripts homolog B | 60 | 1.00E-07 |
| 275 | pgc_singlet01427 | ref|NP_989471.1| ribosomal protein L36 [Gallus gallus] | 103 | 8.00E-21 |
| 276 | pgc_singlet01429 | gb|AAH73237.1| Unknown (protein for MGC:80569) [Xenopus laevis] | 64 | 3.00E-09 |
| 277 | pgc_singlet01455 | ref|NP_997213.1| hypothetical protein LOC152519 [Homo sapiens] | 290 | 5.00E-77 |
| 278 | pgc_singlet01459 | dbj|BAA22950.1| Na+-glucose cotransporter type 1 (SGLT-1)-like protein | 560 | e-162 |
| 279 | pgc_singlet01468 | ref|XP_230874.1| similar to Molybdenum cofactor synthesis protein | 216 | 8.00E-55 |
| 280 | pgc_singlet01479 | ref|NP_078954.3| hypothetical protein FLJ23441 [Homo sapiens] | 384 | e-105 |
| 281 | pgc_singlet01483 | gb|AAQ02399.1| hypothetical protein FLJ14813 [synthetic construct] | 169 | 1.00E-40 |
| 282 | pgc_singlet01503 | ref|NP_989472.1| ribosomal protein L22 [Gallus gallus] | 118 | 3.00E-25 |
| 283 | pgc_singlet01504 | gb|AAP36943.1| Homo sapiens cold inducible RNA binding protein | 179 | 2.00E-43 |
| 284 | pgc_singlet01505 | dbj|BAA11483.2| KIAA0166 [Homo sapiens] | 93 | 3.00E-27 |
| 285 | pgc_singlet01508 | dbj|BAA76800.1| KIAA0956 protein [Homo sapiens] | 528 | e-148 |
| 286 | pgc_singlet01510 | gb|AAH58948.1| Dlgap4 protein [Mus musculus] | 311 | 3.00E-83 |
| 287 | pgc_singlet01518 | gb|AAG45219.1| retinoblastoma tumor suppressor [Gallus gallus] | 46 | 1.00E-15 |
| 288 | pgc_singlet01519 | ref|NP_036142.2| exonuclease 1 [Mus musculus] | 378 | e-103 |
| 289 | pgc_singlet01527 | emb|CAF90690.1| unnamed protein product [Tetraodon nigroviridis] | 116 | 4.00E-51 |
| 290 | pgc_singlet01536 | gb|AAR96048.1| adiponectin receptor-1 [Bos taurus] | 118 | 2.00E-25 |
| 291 | pgc_singlet01544 | gb|AAP36943.1| Homo sapiens cold inducible RNA binding protein | 121 | 9.00E-27 |
| 292 | pgc_singlet01545 | dbj|BAA86605.2| KIAA1291 protein [Homo sapiens] | 53 | 2.00E-07 |
| 293 | pgc_singlet01546 | gb|AAQ88449.1| ASEL436 [Homo sapiens] | 84 | 6.00E-15 |
| 294 | pgc_singlet01555 | ref|NP_003015.2| intersectin 1 isoform ITSN-l; SH3 domain protein | 481 | e-134 |
| 295 | pgc_singlet01559 | ref|NP_989013.1| hypothetical protein MGC76042 | 98 | 4.00E-19 |
| 296 | pgc_singlet01560 | ref|NP_990216.1| ezrin [Gallus gallus] | 55 | 4.00E-06 |
| 297 | pgc_singlet01568 | gb|AAR28760.1| DNA-dependent protein kinase catalytic subunit | 124 | 6.00E-27 |
| 298 | pgc_singlet01581 | ref|NP_056607.1| F-box only protein 18 [Mus musculus] | 288 | 3.00E-76 |
| 299 | pgc_singlet01583 | emb|CAA52982.1| PHAPII (Putative HLA DR Associated Protein II) | 349 | 6.00E-95 |
| 300 | pgc_singlet01589 | gb|AAF76888.1| vitiligo-associated protein VIT-1 [Homo sapiens] | 174 | 5.00E-42 |
| 301 | pgc_singlet01597 | ref|NP_612419.1| chemokine-like factor superfamily 7 isoform a | 126 | 1.00E-27 |
| 302 | pgc_singlet01618 | sp|Q60547|SCP3_MESAU Synaptonemal complex protein 3 (SCP-3 protein) | 59 | 3.00E-07 |
| 303 | pgc_singlet01621 | emb|CAG07254.1| unnamed protein product [Tetraodon nigroviridis] | 247 | 5.00E-64 |
| 304 | pgc_singlet01626 | ref|NP_113980.1| sperm antigen 4 [Rattus norvegicus] | 169 | 1.00E-40 |
| 305 | pgc_singlet01639 | dbj|BAB21885.1| hypothetical protein [Macaca fascicularis] | 112 | 2.00E-23 |
| 306 | pgc_singlet01648 | dbj|BAA86563.1| KIAA1249 protein [Homo sapiens] | 315 | 1.00E-84 |
| 307 | pgc_singlet01650 | ref|NP_002873.1| RAN binding protein 1 [Homo sapiens] | 307 | 3.00E-82 |
| 308 | pgc_singlet01653 | ref|NP_989805.1| type IIb sodium phosphate cotransporter | 103 | 6.00E-21 |
| 309 | pgc_singlet01658 | ref|XP_126866.5| kinase D-interacting substance of 220 kDa | 530 | e-149 |
| 310 | pgc_singlet01662 | ref|NP_990393.1| receptor-associated protein [Gallus gallus] | 563 | e-159 |
| 311 | pgc_singlet01670 | ref|NP_064706.1| MAX binding protein; Max-interacting protein | 91 | 6.00E-17 |
| 312 | pgc_singlet01689 | ref|XP_131572.3| eukaryotic translation initiation factor 2B, subunit 3 | 150 | 3.00E-35 |
| 313 | pgc_singlet01690 | emb|CAE51322.1| basement membrane-specific heparan sulfate protein | 526 | e-148 |
| 314 | pgc_singlet01691 | ref|NP_989520.1| uracil-DNA glycosylase [Gallus gallus] | 398 | e-109 |
| 315 | pgc_singlet01698 | ref|NP_113961.1| RNA polymerase 1-2; RNA polymerase I (127 kDa subunit) | 208 | 2.00E-54 |
| 316 | pgc_singlet01699 | dbj|BAC57944.1| Werner helicase interacting protein [Gallus gallus] | 198 | 2.00E-49 |
| 317 | pgc_singlet01719 | ref|XP_290809.2| TAF4b RNA polymerase II, TATA box binding protein | 201 | 4.00E-60 |
| 318 | pgc_singlet01735 | sp|Q04791|SASB_ANAPL Fatty acyl-CoA hydrolase precursor, medium chain | 310 | 6.00E-83 |
| 319 | pgc_singlet01737 | emb|CAA56563.1| protein-tyrosine-phosphatase [Homo sapiens] | 289 | 1.00E-76 |
| 320 | pgc_singlet01740 | ref|NP_990240.1| GTP-binding protein [Gallus gallus] | 355 | 1.00E-96 |
| 321 | pgc_singlet01742 | sp|Q91642|PEPE_XENLA Alpha-aspartyl dipeptidase (Asp-specific dipeptidase) | 396 | e-109 |
| 322 | pgc_singlet01745 | gb|AAH33429.1| RIKEN cDNA 5730494N06 [Mus musculus] | 186 | 1.00E-45 |
| 323 | pgc_singlet01752 | gb|AAC60281.1| unknown [Gallus gallus] | 74 | 1.00E-16 |
| 324 | pgc_singlet01753 | ref|XP_371632.1| hypothetical protein XP_376222 [Homo sapiens] | 98 | 3.00E-19 |
| 325 | pgc_singlet01757 | ref|NP_579846.1| PR domain containing 4 [Rattus norvegicus] | 390 | e-107 |
| 326 | pgc_singlet01768 | gb|AAH57708.1| MGC68839 protein [Xenopus laevis] | 350 | 3.00E-95 |
| 327 | pgc_singlet01770 | ref|NP_989839.1| polycomblike 2 [Gallus gallus] | 465 | e-130 |
| 328 | pgc_singlet01772 | sp|O42412|IOD3_CHICK Type III iodothyronine deiodinase (Type-III 5'deiodinase) | 387 | e-106 |
| 329 | pgc_singlet01774 | ref|NP_034938.1| matrix metalloproteinase 24; Membrane type 5-MMP | 379 | e-104 |
| 330 | pgc_singlet01775 | gb|AAP88896.1| aldo-keto reductase family 7, member A2 | 127 | 4.00E-28 |
| 331 | pgc_singlet01784 | ref|NP_950243.1| THAP domain containing, apoptosis associated protein 1 | 270 | 7.00E-71 |
| 332 | pgc_singlet01797 | ref|NP_660350.2| pleckstrin homology domain containing, family K member 1 | 288 | 2.00E-76 |
| 333 | pgc_singlet01800 | ref|NP_006808.1| endoplasmic reticulum protein 29 precursor; end... | 65 | 2.00E-14 |
| 334 | pgc_singlet01805 | ref|XP_231122.2| similar to nima -related kinase (1C941) | 56 | 7.00E-10 |
| 335 | pgc_singlet01811 | dbj|BAA88337.1| ORF2 [Platemys spixii] | 70 | 2.00E-24 |
| 336 | pgc_singlet01819 | dbj|BAA88337.1| ORF2 [Platemys spixii] | 102 | 4.00E-45 |
| 337 | pgc_singlet01827 | dbj|BAB55205.1| unnamed protein product [Homo sapiens] | 379 | e-104 |
| 338 | pgc_singlet01840 | ref|NP_005433.2| eomesodermin; t box, brain, 2; eomesodermin | 494 | e-138 |
| 339 | pgc_singlet01843 | ref|XP_236703.2| similar to Eomesodermin homolog | 434 | e-120 |
| 340 | pgc_singlet01845 | dbj|BAA91559.1| unnamed protein product [Homo sapiens] | 148 | 2.00E-34 |
| 341 | pgc_singlet01853 | pir||S49172 translation initiation factor eIF-4 gamma - human (fragment) | 115 | 2.00E-24 |
| 342 | pgc_singlet01887 | ref|NP_989833.1| hypothetical protein LOC395163 [Gallus gallus] | 372 | e-101 |
| 343 | pgc_singlet01889 | dbj|BAA31646.2| KIAA0671 protein [Homo sapiens] | 561 | e-158 |
| 344 | pgc_singlet01896 | ref|NP_000529.1| regulatory factor X-associated protein; RFX-ass... | 127 | 6.00E-28 |
| 345 | pgc_singlet01900 | ref|NP_060661.2| regulator of chromosome condensation (RCC1) and BTB (POZ) domain | 525 | e-147 |
| 346 | pgc_singlet01904 | dbj|BAA88337.1| ORF2 [Platemys spixii] | 71 | 4.00E-11 |
| 347 | pgc_singlet01907 | ref|NP_990047.1| norepinephrine transporter [Gallus gallus] | 246 | 1.00E-63 |
| 348 | pgc_singlet01908 | ref|NP_055026.1| gamma-aminobutyric acid (GABA) A receptor, pi | 176 | 1.00E-42 |
| 349 | pgc_singlet01915 | ref|NP_653147.1| flavin containing monooxygenase 4 | 167 | 2.00E-40 |
| 350 | pgc_singlet01925 | gb|AAC50296.1| band 7.2b stomatin | 439 | e-122 |
| 351 | pgc_singlet01926 | gb|AAA90972.1| kinesin light chain | 277 | 4.00E-73 |
| 352 | pgc_singlet01934 | pir||A38331 LEP100 protein precursor - chicken | 66 | 2.00E-09 |
| 353 | pgc_singlet01935 | gb|AAA49217.1| alpha-enolase/tau-crystallin | 90 | 1.00E-16 |
| 354 | pgc_singlet01940 | ref|NP_003309.2| TTK protein kinase [Homo sapiens] | 270 | 1.00E-75 |
| 355 | pgc_singlet01953 | ref|NP_542964.1| solute carrier family 21 (organic anion transporter), member 9 | 288 | 1.00E-76 |
| 356 | pgc_singlet01962 | dbj|BAA23693.3| KIAA0397 protein [Homo sapiens] | 330 | 6.00E-89 |
| 357 | pgc_singlet01969 | pdb|1FQV|A Chain A, Insights Into Scf Ubiquitin Ligases from The Structure Of The Skp1-Skp2 Complex | 343 | 6.00E-93 |
| 358 | pgc_singlet01978 | ref|NP_796085.2| RIKEN cDNA E230015L20 gene [Mus musculus] | 62 | 2.00E-08 |
| 359 | pgc_singlet01993 | gb|AAK56086.1| copine 1 protein [Mus musculus] | 241 | 2.00E-62 |
| 360 | pgc_singlet01994 | dbj|BAC75408.1| IQ motif containing GTPase activating protein 2 | 363 | 6.00E-99 |
| 361 | pgc_singlet01995 | ref|NP_004438.2| epidermal growth factor receptor pathway substrate 8 | 276 | 9.00E-73 |
| 362 | pgc_singlet01998 | gb|AAH59996.1| MGC68498 protein [Xenopus laevis] | 392 | e-108 |
| 363 | pgc_singlet02005 | ref|NP_005745.1| Ras-GTPase-activating protein SH3-domain-binding protein | 229 | 1.00E-58 |
| 364 | pgc_singlet02007 | pir||A38331 LEP100 protein precursor - chicken | 285 | 1.00E-75 |
| 365 | pgc_singlet02014 | ref|NP_060944.2| uncharacterized hypothalamus protein HT013 | 79 | 2.00E-13 |
| 366 | pgc_singlet02020 | ref|NP_653199.2| hypothetical protein MGC24976 [Homo sapiens] | 325 | 1.00E-87 |
| 367 | pgc_singlet02029 | gb|AAF18974.1| myosin regulatory light chain interacting protein | 150 | 5.00E-35 |
| 368 | pgc_singlet02030 | gb|AAC72372.1| succinate dehydrogenase Ip subunit [Gallus gallus] | 487 | e-136 |
| 369 | pgc_singlet02038 | ref|NP_031401.1| TAR DNA binding protein; TAR DNA-binding protein | 50 | 7.00E-13 |
| 370 | pgc_singlet02043 | dbj|BAB15394.1| unnamed protein product [Homo sapiens] | 322 | 8.00E-87 |
| 371 | pgc_singlet02058 | ref|NP_446316.1| valosin-containing protein [Rattus norvegicus] | 605 | e-172 |
| 372 | pgc_singlet02061 | ref|NP_056234.1| adlican [Homo sapiens] | 540 | e-152 |
| 373 | pgc_singlet02062 | emb|CAG09960.1| unnamed protein product [Tetraodon nigroviridis] | 162 | 7.00E-54 |
| 374 | pgc_singlet02063 | dbj|BAA88337.1| ORF2 [Platemys spixii] | 99 | 8.00E-39 |
| 375 | pgc_singlet02066 | sp|P14629|XPG_XENLA DNA-repair protein complementing XP-G cells homolog | 127 | 6.00E-28 |
| 376 | pgc_singlet02071 | ref|NP_002601.1| pyruvate dehydrogenase kinase, isoenzyme 1 | 63 | 1.00E-08 |
| 377 | pgc_singlet02074 | ref|XP_093839.8| KIAA0826 protein [Homo sapiens] | 74 | 7.00E-12 |
| 378 | pgc_singlet02085 | ref|NP_033112.1| RNA polymerase I polypeptide B | 96 | 9.00E-29 |
| 379 | pgc_singlet02101 | ref|NP_989767.1| 5'-nucleotidase [Gallus gallus] | 214 | 8.00E-55 |
| 380 | pgc_singlet02103 | emb|CAB66826.1| hypothetical protein [Homo sapiens] | 348 | 1.00E-94 |
| 381 | pgc_singlet02112 | ref|NP_989985.1| CocoaCrisp [Gallus gallus] | 166 | 1.00E-39 |
| 382 | pgc_singlet02115 | gb|AAH52713.1| Cyfip1 protein [Mus musculus] | 588 | e-167 |
| 383 | pgc_singlet02121 | ref|NP_848699.1| septin 10 isoform 2 [Homo sapiens] | 176 | 1.00E-42 |
| 384 | pgc_singlet02123 | gb|AAH74187.1| Unknown (protein for MGC:82074) [Xenopus laevis] | 100 | 2.00E-20 |
| 385 | pgc_singlet02128 | dbj|BAA92076.1| unnamed protein product [Homo sapiens] | 149 | 9.00E-35 |
| 386 | pgc_singlet02133 | gb|AAR98743.1| ASPM [Aotus sp. PDE-2004] | 204 | 3.00E-51 |
| 387 | pgc_singlet02137 | ref|NP_060926.2| ELL associated factor 2; uncharacterized bone marrow protein BM040 | 311 | 3.00E-83 |
| 388 | pgc_singlet02139 | ref|XP_215249.2| similar to putative mitochondrial solute carrier | 193 | 2.00E-49 |
| 389 | pgc_singlet02140 | gb|AAM94900.1| membrane protein SB87 precursor [Homo sapiens] | 368 | e-113 |
| 390 | pgc_singlet02142 | ref|XP_371059.1| similar to FALZ protein [Homo sapiens] | 48 | 2.00E-10 |
| 391 | pgc_singlet02147 | ref|NP_990126.1| Dach2 protein [Gallus gallus] | 136 | 1.00E-30 |
| 392 | pgc_singlet02150 | ref|NP_057399.1| GULP, engulfment adaptor PTB domain containing 1 | 361 | 2.00E-98 |
| 393 | pgc_singlet02154 | ref|XP_132230.3| RIKEN cDNA 1810024J13 [Mus musculus] | 263 | 1.00E-73 |
| 394 | pgc_singlet02158 | gb|AAD49808.1| gag/env fusion protein [Gallus gallus] | 104 | 2.00E-29 |
| 395 | pgc_singlet02161 | sp|P53619|COPD_BOVIN Coatomer delta subunit (Delta-coat protein) | 301 | 3.00E-80 |
| 396 | pgc_singlet02164 | ref|NP_036142.2| exonuclease 1 [Mus musculus] | 404 | e-111 |
| 397 | pgc_singlet02175 | ref|ZP_00262451.1| hypothetical protein Pflu02005197 | 309 | 1.00E-82 |
| 398 | pgc_singlet02176 | dbj|BAC33313.1| unnamed protein product [Mus musculus] | 80 | 1.00E-13 |
| 399 | pgc_singlet02186 | gb|AAH47564.1| TRIM6 protein [Homo sapiens] | 86 | 1.00E-15 |
| 400 | pgc_singlet02192 | gb|AAC60281.1| unknown [Gallus gallus] | 131 | 8.00E-49 |
| 401 | pgc_singlet02200 | ref|NP_003757.1| beclin 1; beclin 1 (coiled-coil, myosin-like BCL2-interacting protein) | 370 | e-101 |
| 402 | pgc_singlet02201 | sp|Q9ER65|CLS2_MOUSE Calsyntenin-2 precursor > | 498 | e-139 |
| 403 | pgc_singlet02220 | ref|NP_001003.1| ribosomal protein S8; 40S ribosomal protein S8 | 183 | 3.00E-45 |
| 404 | pgc_singlet02225 | pir||T08700 hypothetical protein DKFZp564G013.1 - human (fragment) | 152 | 1.00E-35 |
| 405 | pgc_singlet02246 | gb|AAH44085.1| MGC52712 protein [Xenopus laevis] | 96 | 1.00E-18 |
| 406 | pgc_singlet02247 | gb|AAG45219.1| retinoblastoma tumor suppressor [Gallus gallus] | 59 | 3.00E-10 |
| 407 | pgc_singlet02265 | dbj|BAA74875.2| KIAA0852 protein [Homo sapiens] | 303 | 7.00E-81 |
| 408 | pgc_singlet02266 | dbj|BAC37288.1| unnamed protein product [Mus musculus] | 483 | e-135 |
| 409 | pgc_singlet02268 | gb|AAH43845.1| Flj13912-prov protein [Xenopus laevis] | 145 | 2.00E-33 |
| 410 | pgc_singlet02269 | dbj|BAB21819.1| KIAA1728 protein [Homo sapiens] | 93 | 1.00E-17 |
| 411 | pgc_singlet02271 | gb|AAH62199.1| Glutamate receptor, ionotropic, N-methyl D-aspartate-like 1A | 58 | 4.00E-07 |
| 412 | pgc_singlet02272 | ref|XP_216945.2| similar to hypothetical protein | 59 | 3.00E-07 |
| 413 | pgc_singlet02281 | dbj|BAC26596.1| unnamed protein product [Mus musculus] | 412 | e-114 |
| 414 | pgc_singlet02285 | gb|AAH28834.1| 4933407C03Rik protein [Mus musculus] | 89 | 2.00E-16 |
| 415 | pgc_singlet02290 | ref|NP_620417.1| secretory carrier membrane protein 5 | 336 | 6.00E-91 |
| 416 | pgc_singlet02306 | ref|XP_282904.1| RIKEN cDNA C030003D03 [Mus musculus] | 92 | 2.00E-17 |
| 417 | pgc_singlet02312 | gb|AAH14546.1| Actin-related protein 2 [Homo sapiens] | 272 | 1.00E-71 |
| 418 | pgc_singlet02313 | gb|AAB47133.1| RhoE [Homo sapiens] | 81 | 5.00E-14 |
| 419 | pgc_singlet02319 | ref|NP_003866.1| guanine monophosphate synthetase; guanosine 5'-monophosphate synthase | 393 | e-108 |
| 420 | pgc_singlet02320 | dbj|BAA32483.1| AF-6 [Homo sapiens] | 228 | 2.00E-78 |
| 421 | pgc_singlet02324 | gb|AAH04912.2| BPAG1 protein [Homo sapiens] | 188 | 2.00E-46 |
| 422 | pgc_singlet02332 | gb|AAH49332.1| Zgc:56577 protein [Danio rerio] | 246 | 1.00E-63 |
| 423 | pgc_singlet02338 | ref|NP_074036.1| core-binding factor, beta subunit isoform 1 | 153 | 7.00E-36 |
| 424 | pgc_singlet02340 | gb|AAH10923.1| Cartilage paired-class homeoprotein 1 | 419 | e-116 |
| 425 | pgc_singlet02341 | ref|NP_689758.1| RasGEF domain family, member 1B; GPI-gamma 4 | 462 | e-129 |
| 426 | pgc_singlet02347 | ref|NP_001001767.1| zinc finger protein 313 [Gallus gallus] | 225 | 2.00E-57 |
| 427 | pgc_singlet02351 | pir||I53784 tropomyosin - rat | 162 | 2.00E-38 |
| 428 | pgc_singlet02353 | ref|XP_030669.3| similar to hypothetical protein [Homo sapiens] | 67 | 7.00E-10 |
| 429 | pgc_singlet02356 | ref|XP_239014.2| similar to sporulation-induced transcript 4-associated protein | 154 | 3.00E-36 |
| 430 | pgc_singlet02366 | ref|NP_444305.2| RAS-homolog enriched in brain [Mus musculus] > | 307 | 3.00E-82 |
| 431 | pgc_singlet02379 | ref|NP_077010.1| dolichyl-P-mannose:Man7GlcNAc2-PP-dolichyl mannosyltransferase | 412 | e-125 |
| 432 | pgc_singlet02395 | emb|CAD38819.1| hypothetical protein [Homo sapiens] | 65 | 3.00E-09 |
| 433 | pgc_singlet02396 | sp|Q8CB77|ELA1_MOUSE Transcription elongation factor B polypeptide | 70 | 1.00E-10 |
| 434 | pgc_singlet02398 | ref|NP_149056.1| DMRT-like family B with proline-rich C-terminal, 1 | 74 | 5.00E-12 |
| 435 | pgc_singlet02403 | gb|AAP36947.1| Homo sapiens ubiquitin-conjugating enzyme E2G 1 | 150 | 8.00E-35 |
| 436 | pgc_singlet02410 | ref|NP_700441.1| RIKEN cDNA 4922503N01 [Mus musculus] | 398 | e-109 |
| 437 | pgc_singlet02414 | ref|NP_033318.1| stimulated by retinoic acid gene 8 | 164 | 4.00E-39 |
| 438 | pgc_singlet02419 | ref|NP_080834.2| RIKEN cDNA 6720467C03 [Mus musculus] | 135 | 2.00E-30 |
| 439 | pgc_singlet02421 | ref|NP_776855.1| protein phosphatase 1B (formerly 2C), magnesium dependant, beta isoform | 459 | e-128 |
| 440 | pgc_singlet02436 | gb|AAH68789.1| Unknown (protein for MGC:81339) [Xenopus laevis] | 110 | 8.00E-23 |
| 441 | pgc_singlet02438 | ref|NP_848599.2| hypothetical protein FLJ40427 [Homo sapiens] | 243 | 9.00E-63 |
| 442 | pgc_singlet02439 | pir||MCRB calmodulin - rabbit (tentative sequence) | 69 | 5.00E-24 |
| 443 | pgc_singlet02442 | dbj|BAC02711.1| KIAA2002 protein [Homo sapiens] | 483 | e-135 |
| 444 | pgc_singlet02446 | gb|AAH26860.1| Fgd6 protein [Mus musculus] | 163 | 6.00E-39 |
| 445 | pgc_singlet02472 | ref|XP_340932.1| similar to Dynein intermediate chain 2, axonemal | 366 | e-100 |
| 446 | pgc_singlet02479 | sp|O75165|DJCD_HUMAN DnaJ homolog subfamily C member 13 | 607 | e-172 |
| 447 | pgc_singlet02481 | ref|NP_700473.1| nephrocystin 4; nephroretinin [Mus musculus] | 353 | 3.00E-96 |
| 448 | pgc_singlet02517 | ref|NP_071428.2| SoxLZ/Sox6 leucine zipper binding protein;SoxLZ/Sox6-binding protein Solt | 142 | 2.00E-32 |
| 449 | pgc_singlet02523 | ref|XP_215673.2| similar to solute carrier family 16 | 263 | 8.00E-69 |
| 450 | pgc_singlet02525 | ref|XP_341374.1| similar to chromosome 13 open reading frame 7 | 278 | 2.00E-73 |
| 451 | pgc_singlet02526 | ref|NP_056128.1| Rho guanine nucleotide exchange factor (GEF) 12 | 264 | 4.00E-69 |
| 452 | pgc_singlet02533 | sp|Q9ER72|SYC_MOUSE Cysteinyl-tRNA synthetase | 183 | 1.00E-68 |
| 453 | pgc_singlet02535 | ref|XP_213618.2| similar to Umps protein [Rattus norvegicus] | 316 | 6.00E-85 |
| 454 | pgc_singlet02536 | gb|AAD49808.1| gag/env fusion protein [Gallus gallus] | 175 | 7.00E-53 |
| 455 | pgc_singlet02539 | dbj|BAC41453.1| mKIAA0864 protein [Mus musculus] | 387 | e-112 |
| 456 | pgc_singlet02540 | emb|CAF90270.1| unnamed protein product [Tetraodon nigroviridis] | 150 | 5.00E-35 |
| 457 | pgc_singlet02541 | ref|NP_006422.1| chaperonin containing TCP1, subunit 2 (beta) | 464 | e-136 |
| 458 | pgc_singlet02545 | ref|NP_956651.1| hypothetical protein MGC63992 [Danio rerio] | 60 | 8.00E-08 |
| 459 | pgc_singlet02547 | sp|P48440|OST4_CHICK Dolichyl-diphosphooligosaccharide--protein | 384 | e-105 |
| 460 | pgc_singlet02548 | gb|AAH13409.2| HCNGP protein [Homo sapiens] | 212 | 2.00E-53 |
| 461 | pgc_singlet02559 | ref|NP_996841.1| latent transforming growth factor beta binding protein 1 isoform b | 181 | 3.00E-44 |
| 462 | pgc_singlet02570 | ref|NP_444305.2| RAS-homolog enriched in brain [Mus musculus] | 356 | 6.00E-97 |
| 463 | pgc_singlet02573 | ref|NP_078968.3| hypothetical protein FLJ21069 [Homo sapiens] | 166 | 1.00E-39 |
| 464 | pgc_singlet02574 | sp|Q865S0|RCAS_CANFA Receptor-binding cancer antigen expressed on SiSo cell | 253 | 7.00E-75 |
| 465 | pgc_singlet02576 | gb|AAH28188.1| Unknown (protein for IMAGE:4385301) [Homo sapiens] | 155 | 2.00E-36 |
| 466 | pgc_singlet02593 | ref|NP_080176.1| DEK oncogene (DNA binding) [Mus musculus] | 256 | 6.00E-67 |
| 467 | pgc_singlet02600 | ref|XP_194371.3| RIKEN cDNA 3110041P15 [Mus musculus] | 345 | 7.00E-95 |
| 468 | pgc_singlet02619 | sp|P49585|CTPT_HUMAN Cholinephosphate cytidylyltransferase A | 244 | 1.00E-63 |
| 469 | pgc_singlet02626 | ref|NP_490595.1| cyclin T2 isoform b; cyclin T2a; cyclin T2b | 224 | 4.00E-88 |
| 470 | pgc_singlet02631 | emb|CAB89807.1| unc-93 related protein [Gallus gallus] | 157 | 5.00E-70 |
| 471 | pgc_singlet02633 | sp|P51003|PAP_HUMAN Poly(A) polymerase alpha (PAP) | 607 | e-172 |
| 472 | pgc_singlet02641 | ref|NP_035632.1| syntaxin 3 isoform C; syntaxin 3A; syntaxin 3B | 294 | 2.00E-78 |
| 473 | pgc_singlet02647 | dbj|BAC04504.1| unnamed protein product [Homo sapiens] | 307 | 3.00E-82 |
| 474 | pgc_singlet02654 | ref|NP_598649.1| sphingomyelin phosphodiesterase, acid-like 3B | 268 | 2.00E-70 |
| 475 | pgc_singlet02657 | gb|AAC97371.2| HIWI [Homo sapiens] | 334 | 2.00E-90 |
| 476 | pgc_singlet02662 | ref|XP_341971.1| similar to PTPRF interacting protein alpha 1 isoform b | 120 | 4.00E-26 |
| 477 | pgc_singlet02668 | dbj|BAB84937.1| FLJ00182 protein [Homo sapiens] | 361 | 2.00E-98 |
| 478 | pgc_singlet02672 | ref|NP_989637.1| platelet-derived growth factor alpha polypeptide | 313 | 5.00E-84 |
| 479 | pgc_singlet02675 | gb|AAH06863.1| Fatty acid amide hydrolase [Mus musculus] | 201 | 4.00E-50 |
| 480 | pgc_singlet02681 | ref|NP_990379.1| CDC42 protein [Gallus gallus] | 390 | e-107 |
| 481 | pgc_singlet02691 | dbj|BAA82513.1| p32 subunit of splicing factor SF2 [Gallus gallus] | 335 | 2.00E-90 |
| 482 | pgc_singlet02695 | gb|AAH36023.1| Polo-like kinase 4 [Homo sapiens] | 293 | 7.00E-78 |
| 483 | pgc_singlet02708 | sp|Q93073|Y256_HUMAN Hypothetical protein KIAA0256 | 130 | 3.00E-43 |
| 484 | pgc_singlet02714 | ref|NP_079528.1| Ras association and pleckstrin homology domains 1 isoform 2 | 577 | e-163 |
| 485 | pgc_singlet02717 | gb|AAS07042.1| minus agglutinin [Chlamydomonas reinhardtii] | 62 | 2.00E-08 |
| 486 | pgc_singlet02720 | ref|NP_057622.1| sirtuin 7; sir2-related protein type 7 | 333 | 4.00E-90 |
| 487 | pgc_singlet02722 | ref|XP_344135.1| similar to Nck-associated protein 5 (NAP-5) | 214 | 3.00E-54 |
| 488 | pgc_singlet02743 | ref|XP_098762.6| KIAA1416 protein [Homo sapiens] | 572 | e-162 |
| 489 | pgc_singlet02748 | ref|NP_990473.1| snail like protein [Gallus gallus] | 105 | 1.00E-21 |
| 490 | pgc_singlet02753 | ref|NP_990707.1| kinectin [Gallus gallus] | 63 | 1.00E-08 |
| 491 | pgc_singlet02762 | ref|XP_215365.1| similar to Lactoylglutathione lyase | 327 | 4.00E-88 |
| 492 | pgc_singlet02769 | dbj|BAA74855.2| KIAA0832 protein [Homo sapiens] | 477 | e-133 |
| 493 | pgc_singlet02773 | ref|NP_067676.2| heterogeneous nuclear ribonucleoprotein H3 isoform b | 133 | 1.00E-29 |
| 494 | pgc_singlet02777 | ref|NP_892118.1| disks large-associated protein 4 isoform b | 374 | e-102 |
| 495 | pgc_singlet02779 | ref|NP_004556.1| peroxisomal biogenesis factor 14 | 188 | 3.00E-46 |
| 496 | pgc_singlet02782 | gb|AAH07932.2| FLJ11588 protein [Homo sapiens] | 123 | 5.00E-41 |
| 497 | pgc_singlet02791 | ref|XP_227591.2| similar to RIKEN cDNA E230011A21 gene | 236 | 7.00E-61 |
| 498 | pgc_singlet02792 | emb|CAD39153.1| hypothetical protein [Homo sapiens] | 316 | 8.00E-85 |
| 499 | pgc_singlet02799 | ref|ZP_00320845.1| COG5651: PPE-repeat proteins | 56 | 1.00E-06 |
| 500 | pgc_singlet02804 | gb|AAP36534.1| Homo sapiens STRIN protein [synthetic construct] | 65 | 2.00E-09 |
| 501 | pgc_singlet02805 | ref|NP_443085.2| aarF domain containing kinase 2 | 167 | 2.00E-43 |
| 502 | pgc_singlet02816 | dbj|BAA12105.1| The KIAA0191 gene is expressed ubiquitously. | 456 | e-127 |
| 503 | pgc_singlet02820 | sp|P51893|SAH1_XENLA Adenosylhomocysteinase 1 (S-adenosyl-L-homocysteine hydrolase 1) | 247 | 4.00E-64 |
| 504 | pgc_singlet02828 | ref|NP_004846.3| phosphatidylinositol glycan, class B | 103 | 6.00E-21 |
| 505 | pgc_singlet02829 | ref|NP_075462.1| dynein, axonemal, intermediate polypeptide 2 | 394 | e-120 |
| 506 | pgc_singlet02838 | ref|NP_115678.1| hypothetical protein MGC10911 [Homo sapiens] | 167 | 5.00E-40 |
| 507 | pgc_singlet02848 | ref|NP_990211.1| ATF4 protein [Gallus gallus] | 142 | 2.00E-32 |
| 508 | pgc_singlet02850 | dbj|BAC38347.1| unnamed protein product [Mus musculus] | 56 | 1.00E-06 |
| 509 | pgc_singlet02853 | ref|NP_006691.1| FLN29 gene product [Homo sapiens] | 102 | 1.00E-20 |
| 510 | pgc_singlet02859 | gb|AAH74298.1| Unknown (protein for MGC:84100) [Xenopus laevis] | 419 | e-116 |
| 511 | pgc_singlet02860 | sp|P10042|CRBA_CHICK Beta crystallin A3 | 419 | e-115 |
| 512 | pgc_singlet02869 | dbj|BAA13437.1| KIAA0242 protein [Homo sapiens] | 246 | 8.00E-64 |
| 513 | pgc_singlet02891 | emb|CAF98271.1| unnamed protein product [Tetraodon nigroviridis] | 110 | 5.00E-23 |
| 514 | pgc_singlet02899 | ref|NP_997880.1| SEC6-like 1; SEC6-like 1 (S. cerevisiae) | 56 | 2.00E-06 |
| 515 | pgc_singlet02905 | pir||A47718 reverse transcriptase, pol-like - chicken (fragment) | 181 | 2.00E-44 |
| 516 | pgc_singlet02907 | ref|NP_990035.1| RhoA GTPase [Gallus gallus] | 390 | e-107 |
| 517 | pgc_singlet02913 | gb|AAH39702.1| 6720461J16Rik protein [Mus musculus] | 134 | 4.00E-30 |
| 518 | pgc_singlet02916 | pir||S69890 mitogen inducible gene mig-2 - human | 448 | e-124 |
| 519 | pgc_singlet02918 | emb|CAF97769.1| unnamed protein product [Tetraodon nigroviridis] | 84 | 8.00E-15 |
| 520 | pgc_singlet02925 | ref|NP_078828.1| LAG1 longevity assurance homolog 4 | 315 | 2.00E-84 |
| 521 | pgc_singlet02927 | gb|AAH04276.1| Unknown (protein for IMAGE:3615480) [Homo sapiens] | 77 | 6.00E-13 |
| 522 | pgc_singlet02931 | ref|NP_037454.2| sorting nexin 10 [Homo sapiens] | 249 | 7.00E-65 |
| 523 | pgc_singlet02932 | ref|NP_002145.3| heat shock 70kDa protein 4 isoform a | 504 | e-150 |
| 524 | pgc_singlet02952 | ref|NP_877950.1| hypothetical protein FLJ39155 isoform 2 | 349 | 9.00E-95 |
| 525 | pgc_singlet02964 | ref|NP_057145.1| yippee protein [Homo sapiens] | 101 | 4.00E-20 |
| 526 | pgc_singlet02966 | ref|NP_079435.1| chromosome X open reading frame 21 | 153 | 9.00E-36 |
| 527 | pgc_singlet02968 | gb|AAH05016.1| Unknown (protein for IMAGE:3636175) [Homo sapiens] | 96 | 2.00E-18 |
| 528 | pgc_singlet02978 | gb|AAM94627.1| truncated non-neuronal splice variant nPTB6 | 86 | 1.00E-15 |
| 529 | pgc_singlet02981 | ref|NP_660350.2| pleckstrin homology domain containing, family K member 1 | 258 | 3.00E-67 |
| 530 | pgc_singlet02982 | sp|Q9Y657|SPIN_HUMAN Spindlin (Ovarian cancer-related protein) | 144 | 6.00E-33 |
| 531 | pgc_singlet02990 | dbj|BAA74900.1| KIAA0877 protein [Homo sapiens] | 250 | 4.00E-65 |
| 532 | pgc_singlet02991 | gb|AAR28760.1| DNA-dependent protein kinase catalytic subunit | 72 | 3.00E-11 |
| 533 | pgc_singlet02997 | gb|AAH56054.1| MGC69017 protein [Xenopus laevis] | 69 | 2.00E-10 |
| 534 | pgc_singlet03020 | pir||T08701 hypothetical protein DKFZp564N123.1 - human (fragment) | 93 | 1.00E-17 |
| 535 | pgc_singlet03023 | ref|NP_776185.1| hypothetical protein MGC26717 [Homo sapiens] | 193 | 6.00E-48 |
| 536 | pgc_singlet03026 | ref|NP_990855.1| AIR carboxylase domain [Gallus gallus] | 511 | e-144 |
| 537 | pgc_singlet03030 | gb|AAK92281.1| HIWI [Homo sapiens] | 474 | e-132 |
| 538 | pgc_singlet03038 | ref|NP_777245.1| NADH dehydrogenase (ubiquinone) Fe-S protein 1 | 173 | 9.00E-42 |
| 539 | pgc_singlet03042 | gb|AAC16061.1| poly(A) polymerase II [Gallus gallus] | 443 | e-123 |
| 540 | pgc_singlet03045 | ref|NP_001362.1| dynein, axonemal, heavy polypeptide 8 | 616 | e-175 |
| 541 | pgc_singlet03053 | gb|AAF60061.1| ORFRU4-R [Macaca mulatta rhadinovirus 26-95] | 55 | 4.00E-06 |
| 542 | pgc_singlet03059 | gb|AAH36370.1| Stress 70 protein chaperone, microsome-associated 60kDa, precursor | 191 | 2.00E-47 |
| 543 | pgc_singlet03064 | ref|NP_443097.1| chromosome 20 open reading frame 72 | 156 | 9.00E-59 |
| 544 | pgc_singlet03067 | dbj|BAA86598.1| KIAA1284 protein [Homo sapiens] | 410 | e-120 |
| 545 | pgc_singlet03074 | ref|NP_060853.2| WD repeat domain 33 [Homo sapiens] | 76 | 2.00E-12 |
| 546 | pgc_singlet03076 | dbj|BAA88337.1| ORF2 [Platemys spixii] | 172 | 3.00E-47 |
| 547 | pgc_singlet03083 | pir||A47718 reverse transcriptase, pol-like - chicken (fragment) | 94 | 8.00E-18 |
| 548 | pgc_singlet03084 | ref|NP_766059.1| inter-alpha (globulin) inhibitor H5 | 338 | 1.00E-91 |
| 549 | pgc_singlet03088 | ref|NP_005648.1| tumor protein p53 binding protein, 1; tumor protein 53-binding protein 1 | 377 | e-103 |
| 550 | pgc_singlet03090 | dbj|BAA88337.1| ORF2 [Platemys spixii] | 42 | 4.00E-06 |
| 551 | pgc_singlet03095 | gb|AAH43805.1| MGC64475 protein [Xenopus laevis] | 228 | 2.00E-58 |
| 552 | pgc_singlet03096 | ref|NP_067310.1| U2 small nuclear ribonucleoprotein B | 296 | 7.00E-85 |
| 553 | pgc_singlet03097 | ref|NP_689637.1| hypothetical protein FLJ39827 [Homo sapiens] | 103 | 6.00E-21 |
| 554 | pgc_singlet03098 | gb|AAF87973.1| early response to neural induction ERNI | 90 | 1.00E-16 |
| 555 | pgc_singlet03101 | dbj|BAC30928.1| unnamed protein product [Mus musculus] | 82 | 3.00E-14 |
| 556 | pgc_singlet03103 | emb|CAF98039.1| unnamed protein product [Tetraodon nigroviridis] | 428 | e-118 |
| 557 | pgc_singlet03105 | ref|NP_796316.1| TAF5 RNA polymerase II, TATA box binding protein | 144 | 5.00E-33 |
| 558 | pgc_singlet03107 | sp|O19049|ROK_RABIT Heterogeneous nuclear ribonucleoprotein K | 61 | 7.00E-08 |
| 559 | pgc_singlet03110 | dbj|BAB18155.1| hypothetical protein [Macaca fascicularis] | 216 | 8.00E-55 |
| 560 | pgc_singlet03114 | ref|NP_001001528.1| amylase, alpha, hepatic [Gallus gallus] | 425 | e-118 |
| 561 | pgc_singlet03121 | pir||T01371 hypothetical protein 327024.1 - human | 162 | 9.00E-39 |
| 562 | pgc_singlet03124 | pir||T46292 hypothetical protein DKFZp434E0610.1 - human (fragment) | 301 | 2.00E-80 |
| 563 | pgc_singlet03126 | dbj|BAC87304.1| unnamed protein product [Homo sapiens] | 90 | 1.00E-16 |
| 564 | pgc_singlet03129 | ref|XP_371268.1| similar to 1700028K03 protein [Homo sapiens] | 207 | 3.00E-52 |
| 565 | pgc_singlet03134 | ref|NP_296373.1| F-box only protein 21 isoform 1 | 486 | e-137 |
| 566 | pgc_singlet03136 | ref|XP_110248.2| similar to F-box only protein 11 [Mus musculus] | 217 | 4.00E-55 |
| 567 | pgc_singlet03146 | dbj|BAC04829.1| unnamed protein product [Homo sapiens] | 603 | e-171 |
| 568 | pgc_singlet03148 | ref|NP_766578.1| pantothenate kinase 4 [Mus musculus] | 87 | 6.00E-16 |
| 569 | pgc_singlet03150 | dbj|BAA76791.2| KIAA0947 protein [Homo sapiens] | 84 | 5.00E-15 |
| 570 | pgc_singlet03151 | gb|AAH28742.1| KIAA1340 protein [Homo sapiens] | 373 | e-102 |
| 571 | pgc_singlet03157 | ref|NP_999225.1| esterase D [Sus scrofa] | 300 | 6.00E-80 |
| 572 | pgc_singlet03163 | ref|NP_653199.2| hypothetical protein MGC24976 [Homo sapiens] | 514 | e-156 |
| 573 | pgc_singlet03167 | ref|NP_075954.1| beta-catenin-interacting protein ICAT | 148 | 3.00E-34 |
| 574 | pgc_singlet03168 | sp|P24588|AKA5_HUMAN A-kinase anchor protein 5 | 65 | 5.00E-09 |
| 575 | pgc_singlet03179 | ref|NP_056375.1| optic atrophy 1 isoform 1 [Homo sapiens] | 229 | 1.00E-58 |
| 576 | pgc_singlet03189 | emb|CAC28351.1| Putative Chromatin modulator [Homo sapiens] | 489 | e-137 |
| 577 | pgc_singlet03199 | ref|NP_444305.2| RAS-homolog enriched in brain [Mus musculus] | 356 | 5.00E-97 |
| 578 | pgc_singlet03203 | ref|NP_006132.1| dynein, cytoplasmic, light intermediate polypeptide 2 | 305 | 1.00E-81 |
| 579 | pgc_singlet03205 | emb|CAG10574.1| unnamed protein product [Tetraodon nigroviridis] | 179 | 1.00E-43 |
| 580 | pgc_singlet03210 | gb|AAR11374.1| TEM5-like protein [Homo sapiens] | 274 | 3.00E-72 |
| 581 | pgc_singlet03215 | ref|NP_060217.1| multiple ankyrin repeats, single KH-domain protein isoform 2 | 281 | 2.00E-74 |
| 582 | pgc_singlet03218 | dbj|BAA88337.1| ORF2 [Platemys spixii] | 118 | 3.00E-25 |
| 583 | pgc_singlet03220 | ref|NP_990417.1| ferritin H chain [Gallus gallus] | 336 | 3.00E-91 |
| 584 | pgc_singlet03221 | gb|AAR28760.1| DNA-dependent protein kinase catalytic subunit | 59 | 3.00E-08 |
| 585 | pgc_singlet03222 | ref|NP_079699.1| RIKEN cDNA 1110059E24 [Mus musculus] | 201 | 3.00E-50 |
| 586 | pgc_singlet03235 | pir||I50209 reverse transcriptase VTGIII, pol-like - chicken | 76 | 7.00E-24 |
| 587 | pgc_singlet03238 | ref|NP_852085.1| hypothetical protein 9030012M21 [Mus musculus] | 129 | 1.00E-51 |
| 588 | pgc_singlet03251 | gb|AAH70132.1| Unknown (protein for IMAGE:30369720) [Homo sapiens] | 386 | e-106 |
| 589 | pgc_singlet03254 | dbj|BAB71045.1| unnamed protein product [Homo sapiens] | 536 | e-151 |
| 590 | pgc_singlet03258 | emb|CAG09396.1| unnamed protein product [Tetraodon nigroviridis] | 102 | 2.00E-20 |
| 591 | pgc_singlet03270 | ref|NP_004804.1| peroxisomal biogenesis factor 16 isoform 1 | 275 | 6.00E-74 |
| 592 | pgc_singlet03274 | sp|Q9WVR1|INP5_RAT 72 kDa inositol polyphosphate 5-phosphatase | 486 | e-136 |
| 593 | pgc_singlet03279 | ref|NP_002873.1| RAN binding protein 1 [Homo sapiens] | 307 | 4.00E-82 |
| 594 | pgc_singlet03281 | gb|AAH37432.1| Unknown (protein for IMAGE:2648077) [Mus musculus] | 437 | e-121 |
| 595 | pgc_singlet03298 | ref|XP_235549.2| similar to FLJ20699 protein [Rattus norvegicus] | 428 | e-118 |
| 596 | pgc_singlet03299 | ref|NP_950197.1| similar to KIAA0635 gene product [Mus musculus]... | 411 | e-113 |
| 597 | pgc_singlet03304 | ref|XP_220057.2| similar to expressed sequence AW210596 | 234 | 2.00E-60 |
| 598 | pgc_singlet03305 | gb|AAH67294.1| ZNF6 protein [Homo sapiens] | 362 | e-102 |
| 599 | pgc_singlet03310 | gb|AAH44085.1| MGC52712 protein [Xenopus laevis] | 96 | 1.00E-18 |
| 600 | pgc_singlet03317 | ref|NP_990308.1| collapsin [Gallus gallus] | 154 | 5.00E-36 |
| 601 | pgc_singlet03323 | gb|AAH04679.1| Hnrpr protein [Mus musculus] | 122 | 2.00E-26 |
| 602 | pgc_singlet03330 | gb|AAG31060.1| G-protein B1 subunit [Ambystoma tigrinum] | 489 | e-137 |
| 603 | pgc_singlet03334 | pir||A47718 reverse transcriptase, pol-like - chicken (fragment) | 82 | 2.00E-14 |
| 604 | pgc_singlet03337 | ref|XP_233728.2| similar to FLJ00414 protein [Rattus norvegicus] | 96 | 3.00E-20 |
| 605 | pgc_singlet03342 | ref|NP_004806.2| PTPL1-associated RhoGAP 1 [Homo sapiens] | 131 | 6.00E-32 |
| 606 | pgc_singlet03343 | ref|NP_003617.1| PTPRF interacting protein alpha 1 isoform b | 259 | 9.00E-68 |
| 607 | pgc_singlet03358 | ref|NP_061027.1| low density lipoprotein-related protein 1B | 312 | 9.00E-84 |
| 608 | pgc_singlet03371 | ref|XP_342853.1| similar to RIKEN cDNA 2410141F18 | 301 | 2.00E-80 |
| 609 | pgc_singlet03420 | ref|XP_341842.1| similar to RIKEN cDNA 2700023B17 | 349 | e-106 |
| 610 | pgc_singlet03422 | ref|XP_291816.4| otogelin [Homo sapiens] | 333 | 7.00E-90 |
| 611 | pgc_singlet03427 | ref|XP_237242.2| similar to ATP-binding cassette, sub-family A, member 12 isoform a | 417 | e-115 |
| 612 | pgc_singlet03431 | ref|NP_000103.1| solute carrier family 26 member 2 | 165 | 2.00E-39 |
| 613 | pgc_singlet03433 | emb|CAF88807.1| unnamed protein product [Tetraodon nigroviridis] | 217 | 5.00E-55 |
| 614 | pgc_singlet03435 | ref|NP_989881.1| minibrain protein kinase [Gallus gallus] | 170 | 7.00E-41 |
| 615 | pgc_singlet03440 | ref|NP_033180.1| semaphorin 5A; semaphorin F; M-Sema D | 533 | e-150 |
| 616 | pgc_singlet03447 | gb|AAF66429.1| envelope protein [Avian leukosis virus] | 407 | e-125 |
| 617 | pgc_singlet03448 | ref|NP_004136.2| myosin IXB [Homo sapiens] | 399 | e-110 |
| 618 | pgc_singlet03466 | ref|NP_700356.2| leucine-rich repeats and immunoglobulin-like domains 3 | 92 | 3.00E-17 |
| 619 | pgc_singlet03470 | sp|Q61077|FI14_MOUSE Fibroblast growth factor inducible protein | 65 | 4.00E-09 |
| 620 | pgc_singlet03479 | ref|NP_001780.2| cell division cycle 25A isoform a | 183 | 6.00E-45 |
| 621 | pgc_singlet03487 | ref|NP_659095.1| RIKEN cDNA 0910001A06 [Mus musculus] | 362 | e-103 |
| 622 | pgc_singlet03493 | gb|AAM93271.1| premature ovarian failure 1B protein [Mus musculus] | 119 | 2.00E-25 |
| 623 | pgc_singlet03498 | gb|AAH46976.1| Htf9c protein [Mus musculus] | 216 | 1.00E-54 |
| 624 | pgc_singlet03501 | sp|P51893|SAH1_XENLA Adenosylhomocysteinase 1 | 417 | e-115 |
| 625 | pgc_singlet03508 | emb|CAA09178.1| intestinal Muc 2-like protein [Mus musculus] | 390 | e-116 |
| 626 | pgc_singlet03512 | sp|Q06438|PEX2_CRIGR Peroxisome assembly factor-1 (PAF-1) | 218 | 2.00E-55 |
| 627 | pgc_singlet03514 | ref|XP_231116.1| similar to RIKEN cDNA 2900073H19 | 55 | 3.00E-06 |
| 628 | pgc_singlet03517 | pir||T00067 hypothetical protein KIAA0453 - human (fragment) | 566 | e-160 |
| 629 | pgc_singlet03521 | ref|NP_032284.1| hematological and neurological expressed sequence 1 | 149 | 1.00E-34 |
| 630 | pgc_singlet03529 | ref|XP_375305.1| similar to TSG118.1 protein [Homo sapiens] | 144 | 5.00E-33 |
| 631 | pgc_singlet03537 | ref|NP_996745.1| vav 3 oncogene [Gallus gallus] | 407 | e-112 |
| 632 | pgc_singlet03542 | ref|XP_356329.1| similar to Pro-Pol-dUTPase polyprotein; RNaseH | 94 | 7.00E-18 |
| 633 | pgc_singlet03545 | emb|CAB66540.1| hypothetical protein [Homo sapiens] | 235 | 1.00E-60 |
| 634 | pgc_singlet03552 | dbj|BAA34387.1| G-protein-activated inwardly rectifying potassium channel subunit 4 | 545 | e-156 |
| 635 | pgc_singlet03555 | emb|CAD39063.1| hypothetical protein [Homo sapiens] | 516 | e-145 |
| 636 | pgc_singlet03558 | ref|NP_776364.1| methylmalonyl Coenzyme A mutase [Bos taurus] | 152 | 9.00E-36 |
| 637 | pgc_singlet03562 | dbj|BAB27829.1| unnamed protein product [Mus musculus] | 151 | 3.00E-35 |
| 638 | pgc_singlet03564 | ref|NP_848660.2| hypothetical protein MGC35555 [Homo sapiens] | 410 | e-113 |
| 639 | pgc_singlet03565 | gb|AAC60281.1| unknown [Gallus gallus] | 223 | e-111 |
| 640 | pgc_singlet03572 | ref|NP_033551.1| wingless-related MMTV integration site 5B | 236 | 7.00E-61 |
| 641 | pgc_singlet03574 | pir||A57277 beta-3 endonexin, long splice form - human | 124 | 6.00E-27 |
| 642 | pgc_singlet03576 | dbj|BAA85386.1| GCF2 fusion protein [Homo sapiens] | 353 | 6.00E-96 |
| 643 | pgc_singlet03580 | tpg|DAA00004.1| TPA: pantothenate kinase 2; PANK2 [Homo sapiens] | 245 | 2.00E-63 |
| 644 | pgc_singlet03588 | dbj|BAC97927.1| mKIAA0333 protein [Mus musculus] | 284 | 3.00E-75 |
| 645 | pgc_singlet03599 | ref|XP_218601.2| similar to hypothetical protein FLJ23311 | 66 | 1.00E-09 |
| 646 | pgc_singlet03613 | ref|NP_004775.1| N-deacetylase/N-sulfotransferase (heparan glucosaminyl) 3 | 492 | e-138 |
| 647 | pgc_singlet03618 | gb|AAC60281.1| unknown [Gallus gallus] | 174 | 4.00E-42 |
| 648 | pgc_singlet03630 | ref|NP_033376.1| testis nuclear RNA binding proteiin | 173 | 9.00E-42 |
| 649 | pgc_singlet03635 | ref|NP_989880.1| translational eukaryotic inititation factor 4AII | 392 | e-108 |
| 650 | pgc_singlet03640 | dbj|BAA88337.1| ORF2 [Platemys spixii] | 86 | 2.00E-15 |
| 651 | pgc_singlet03647 | ref|NP_990435.1| aminopeptidase H [Gallus gallus] | 347 | 4.00E-94 |
| 652 | pgc_singlet03656 | ref|XP_358763.1| RIKEN cDNA 2700045P11 [Mus musculus] | 105 | 2.00E-21 |
| 653 | pgc_singlet03657 | dbj|BAB40343.1| cytochrome b [Gallus gallus] | 466 | e-142 |
| 654 | pgc_singlet03668 | ref|NP_003741.1| eukaryotic translation initiation factor 3, subunit 10 theta | 73 | 1.00E-11 |
| 655 | pgc_singlet03677 | gb|AAC36392.1| AR1 [Homo sapiens] | 89 | 1.00E-21 |
| 656 | pgc_singlet03682 | ref|NP_620584.2| SPPL3 protein; presenilin-like protein 4 | 60 | 8.00E-08 |
| 657 | pgc_singlet03683 | gb|AAC69706.1| GTP-specific succinyl-CoA synthetase beta subunit | 103 | 9.00E-21 |
| 658 | pgc_singlet03690 | gb|AAH03931.1| Trpc4ap protein [Mus musculus] | 112 | 2.00E-23 |
| 659 | pgc_singlet03699 | ref|NP_742162.2| hypothetical protein MGC47065 [Mus musculus] | 489 | e-137 |
| 660 | pgc_singlet03706 | emb|CAG14606.1| unnamed protein product [Tetraodon nigroviridis] | 157 | 4.00E-37 |
| 661 | pgc_singlet03707 | gb|AAN28379.1| Abl-interactor 1 [Homo sapiens] | 484 | e-144 |
| 662 | pgc_singlet03708 | emb|CAA51267.1| transformation upregulated nuclear protein | 164 | 4.00E-39 |
| 663 | pgc_singlet03718 | ref|XP_166254.4| odd Oz/ten-m homolog 4 [Homo sapiens] | 167 | 6.00E-40 |
| 664 | pgc_singlet03719 | gb|AAP36892.1| Homo sapiens follicular lymphoma variant translocation 1 | 58 | 5.00E-07 |
| 665 | pgc_singlet03737 | ref|NP_058069.1| neuronal pentraxin 2 [Mus musculus] | 323 | 6.00E-87 |
| 666 | pgc_singlet03749 | ref|NP_004353.1| calmegin [Homo sapiens] | 110 | 8.00E-23 |
| 667 | pgc_singlet03772 | gb|AAH59990.1| MGC68696 protein [Xenopus laevis] | 375 | e-102 |
| 668 | pgc_singlet03774 | gb|AAC24501.1| GDP-D-mannose-4,6-dehydratase [Homo sapiens] | 550 | e-155 |
| 669 | pgc_singlet03781 | ref|NP_872295.1| STRA8 [Homo sapiens] | 193 | 8.00E-48 |
| 670 | pgc_singlet03796 | gb|AAB97010.1| unknown protein IT1 [Homo sapiens] | 230 | 6.00E-59 |
| 671 | pgc_singlet03811 | ref|NP_006404.1| ribonuclease P (30kD) [Homo sapiens] | 193 | 6.00E-48 |
| 672 | pgc_singlet03812 | ref|NP_000218.2| laminin alpha 3 subunit isoform 2 | 227 | 4.00E-58 |
| 673 | pgc_singlet03823 | pdb|1H7U|A Chain A, Hpms2-Atpgs | 301 | 2.00E-80 |
| 674 | pgc_singlet03835 | dbj|BAA84924.1| ABP130 [Homo sapiens] | 339 | 9.00E-92 |
| 675 | pgc_singlet03837 | ref|XP_236481.2| similar to KIAA1417 protein [Rattus norvegicus] | 163 | 7.00E-39 |
| 676 | pgc_singlet03840 | ref|NP_998751.1| mannan-binding lectin associated serine protease 3 | 144 | 4.00E-33 |
| 677 | pgc_singlet03845 | ref|NP_004866.1| RNA polymerase I subunit isoform 2; RNA polymerase I subunit | 72 | 3.00E-11 |
| 678 | pgc_singlet03847 | ref|NP_055862.1| KIAA0090 protein [Homo sapiens] | 322 | 9.00E-87 |
| 679 | pgc_singlet03848 | gb|AAQ91026.1| LRRGT00070 [Rattus norvegicus] | 82 | 4.00E-14 |
| 680 | pgc_singlet03866 | gb|AAH26119.1| Contactin 4, isoform c precursor [Homo sapiens] | 538 | e-151 |
| 681 | pgc_singlet03871 | ref|NP_084505.2| polymerase (RNA) III (DNA directed) polypeptide H | 231 | 3.00E-59 |
| 682 | pgc_singlet03878 | ref|NP_599230.1| Cca3 protein [Rattus norvegicus] | 585 | e-176 |
| 683 | pgc_singlet03911 | ref|XP_233820.2| similar to mouse Son of sevenless 1 | 185 | 2.00E-45 |
| 684 | pgc_singlet03914 | gb|AAG45219.1| retinoblastoma tumor suppressor [Gallus gallus] | 80 | 3.00E-21 |
| 685 | pgc_singlet03919 | prf||2206375A fragile X mental retardation protein | 579 | e-164 |
| 686 | pgc_singlet03928 | sp|Q9H446|RWD1_HUMAN RWD domain containing protein 1 (CGI-24) | 353 | 6.00E-96 |
| 687 | pgc_singlet03942 | ref|NP_001001758.1| cadherin 6B [Gallus gallus] | 363 | 4.00E-99 |
| 688 | pgc_singlet03952 | gb|AAC83166.1| NAD(+)-isocitrate dehydrogenase subunit 1 IDH1-A precursor | 277 | 4.00E-73 |
| 689 | pgc_singlet03964 | ref|NP_006382.1| importin 7; RAN-binding protein 7 | 197 | 3.00E-49 |
| 690 | pgc_singlet03965 | gb|AAH54599.1| Txnrd1 protein [Danio rerio] | 518 | e-145 |
| 691 | pgc_singlet03968 | gb|AAP04415.1| KIF27C [Homo sapiens] | 253 | 6.00E-66 |
| 692 | pgc_singlet03982 | gb|AAH70584.1| Unknown (protein for MGC:81131) [Xenopus laevis] | 422 | e-117 |
| 693 | pgc_singlet03999 | ref|NP_057156.1| CGI-141 protein [Homo sapiens] | 207 | 6.00E-52 |
| 694 | pgc_singlet04018 | ref|NP_989801.1| cyclin S [Gallus gallus] | 102 | 2.00E-20 |
| 695 | pgc_singlet04025 | emb|CAG03679.1| unnamed protein product [Tetraodon nigroviridis] | 356 | 7.00E-97 |
| 696 | pgc_singlet04031 | gb|AAH08312.2| NMT1 protein [Homo sapiens] | 98 | 5.00E-19 |
| 697 | pgc_singlet04034 | gb|AAH03193.1| Leucine rich repeat containing 1 [Homo sapiens] | 314 | 2.00E-84 |
| 698 | pgc_singlet04038 | ref|NP_003015.2| intersectin 1 isoform ITSN-l; SH3 domain protein-1A | 267 | 8.00E-95 |
| 699 | pgc_singlet04044 | ref|NP_002259.1| karyopherin alpha 4; importin alpha 3 | 39 | 7.00E-07 |
| 700 | pgc_singlet04051 | ref|NP_004152.1| RAB1A, member RAS oncogene family | 409 | e-113 |
| 701 | pgc_singlet04057 | ref|NP_000529.1| regulatory factor X-associated protein | 127 | 7.00E-28 |
| 702 | pgc_singlet04060 | ref|NP_057400.1| REV1-like; REV1 protein; REV1 (yeast homolog) | 236 | e-116 |
| 703 | pgc_singlet04080 | dbj|BAA91662.1| unnamed protein product [Homo sapiens] | 75 | 4.00E-12 |
| 704 | pgc_singlet04082 | sp|Q8C0V0|TKL1_MOUSE Serine/threonine-protein kinase tousled-like 1 | 371 | e-101 |
| 705 | pgc_singlet04086 | dbj|BAC41775.1| hypothetical protein [Macaca fascicularis] | 47 | 2.00E-10 |
| 706 | pgc_singlet04103 | dbj|BAB55278.1| unnamed protein product [Homo sapiens] | 354 | 2.00E-96 |
| 707 | pgc_singlet04111 | gb|AAH23187.1| Cdc27 protein [Mus musculus] | 55 | 3.00E-06 |
| 708 | pgc_singlet04115 | ref|XP_345161.1| similar to Polyadenylate binding protein-interacting protein 1 | 154 | 5.00E-36 |
| 709 | pgc_singlet04121 | dbj|BAA20836.1| KIAA0382 [Homo sapiens] | 206 | 9.00E-52 |
| 710 | pgc_singlet04122 | dbj|BAB28933.1| unnamed protein product [Mus musculus] | 107 | 6.00E-22 |
| 711 | pgc_singlet04130 | ref|NP_990379.1| CDC42 protein [Gallus gallus] | 249 | 1.00E-64 |
| 712 | pgc_singlet04142 | sp|Q90935|NEUS_CHICK Neuroserpin precursor (Axonin-2) | 607 | e-172 |
| 713 | pgc_singlet04164 | gb|AAH46242.1| AP1B1 protein [Homo sapiens] | 326 | 6.00E-88 |
| 714 | pgc_singlet04178 | sp|Q95LJ0|PIM1_FELCA Proto-oncogene serine/threonine-protein kinase pim-1 | 182 | 2.00E-44 |
| 715 | pgc_singlet04186 | ref|NP_061840.1| syntrophin, gamma 1; gamma1-syntrophin | 241 | 2.00E-62 |
| 716 | pgc_singlet04196 | ref|XP_370878.1| KIAA2002 protein [Homo sapiens] | 406 | e-113 |
| 717 | pgc_singlet04197 | sp|P12265|BGLR_MOUSE Beta-glucuronidase precursor | 86 | 1.00E-15 |
| 718 | pgc_singlet04209 | ref|XP_375729.1| chromosome 1 open reading frame 34 [Homo sapiens] | 82 | 3.00E-14 |
| 719 | pgc_singlet04213 | ref|NP_037428.2| G-protein signalling modulator 2 | 96 | 2.00E-18 |
| 720 | pgc_singlet04230 | emb|CAB97215.1| T-cell transcription factor-4 long C-terminal isoform 4 | 357 | e-119 |
| 721 | pgc_singlet04231 | pir||A47718 reverse transcriptase, pol-like - chicken (fragment) | 84 | 5.00E-15 |
| 722 | pgc_singlet04239 | dbj|BAC30370.1| unnamed protein product [Mus musculus] | 72 | 2.00E-11 |
| 723 | pgc_singlet04243 | ref|NP_612638.2| histidine triad nucleotide binding protein | 218 | 3.00E-55 |
| 724 | pgc_singlet04244 | sp|P12265|BGLR_MOUSE Beta-glucuronidase precursor | 86 | 1.00E-15 |
| 725 | pgc_singlet04257 | gb|AAH58172.1| Unknown (protein for MGC:67870) [Mus musculus] | 206 | 6.00E-52 |
| 726 | pgc_singlet04273 | pir||E84565 hypothetical protein At2g18540 [imported] | 54 | 6.00E-06 |
| 727 | pgc_singlet04276 | emb|CAA03918.1| spermine synthase [Mus musculus] | 317 | 3.00E-85 |
| 728 | pgc_singlet04278 | ref|NP_006760.1| LIM domain only 4 [Homo sapiens] | 61 | 5.00E-08 |
| 729 | pgc_singlet04291 | gb|AAH58689.1| Unknown (protein for MGC:65672) [Mus musculus] | 94 | 3.00E-18 |
| 730 | pgc_singlet04294 | dbj|BAC97997.1| mKIAA0677 protein [Mus musculus] | 59 | 2.00E-07 |
| 731 | pgc_singlet04308 | ref|NP_990561.1| SCII [Gallus gallus] | 101 | 4.00E-20 |
| 732 | pgc_singlet04309 | emb|CAD38969.1| hypothetical protein [Homo sapiens] | 70 | 8.00E-11 |
| 733 | pgc_singlet04311 | ref|NP_064518.1| 1-acylglycerol-3-phosphate O-acyltransferase 4 | 82 | 3.00E-14 |
| 734 | pgc_singlet04312 | gb|AAA30484.1| cyclophilin-40 | 216 | 1.00E-54 |
| 735 | pgc_singlet04319 | ref|NP_067280.1| Sec61, alpha subunit 2; Sec61 alpha isoform 2 | 125 | 1.00E-27 |
| 736 | pgc_singlet04320 | emb|CAG08420.1| unnamed protein product [Tetraodon nigroviridis] | 105 | 2.00E-21 |
| 737 | pgc_singlet04329 | ref|NP_061911.2| tudor domain containing 4 [Homo sapiens] | 299 | 1.00E-79 |
| 738 | pgc_singlet04333 | ref|NP_036242.1| cysteine-rich hydrophobic domain 2 | 241 | 3.00E-62 |
| 739 | pgc_singlet04341 | ref|NP_766068.1| 5-methyltetrahydrofolate-homocysteine methyltransferase reductase | 256 | 1.00E-66 |
| 740 | pgc_singlet04363 | ref|NP_006073.1| tubulin, alpha, ubiquitous [Homo sapiens] | 228 | e-135 |
| 741 | pgc_singlet04368 | sp|P10999|LAM3_XENLA Lamin L(III) (Lamin B3) | 375 | e-103 |
| 742 | pgc_singlet04384 | ref|XP_344595.1| similar to sox-4 protein - mouse | 80 | 8.00E-14 |
| 743 | pgc_singlet04385 | ref|XP_376472.1| similar to KIAA0319 [Homo sapiens] | 122 | 2.00E-26 |
| 744 | pgc_singlet04387 | ref|NP_080924.1| RIKEN cDNA 4930457P18 [Mus musculus] | 181 | 6.00E-48 |
| 745 | pgc_singlet04396 | emb|CAG09372.1| unnamed protein product [Tetraodon nigroviridis] | 286 | 6.00E-76 |
| 746 | pgc_singlet04400 | ref|NP_061878.2| hypothetical protein FLJ20323 [Homo sapiens] | 98 | 3.00E-19 |
| 747 | pgc_singlet04402 | ref|NP_036520.1| pallidin; pallid (mouse) homolog, pallidin | 156 | 1.00E-36 |
| 748 | pgc_singlet04406 | dbj|BAB84892.1| FLJ00137 protein [Homo sapiens] | 365 | 1.00E-99 |
| 749 | pgc_singlet04410 | ref|NP_004883.1| vesicle trafficking protein sec22b; SEC22, vesicle trafficking protein | 95 | 4.00E-18 |
| 750 | pgc_singlet04411 | emb|CAA10305.1| Xvent-1B protein [Xenopus laevis] | 85 | 3.00E-15 |
| 751 | pgc_singlet04419 | dbj|BAC03973.1| unnamed protein product [Homo sapiens] | 214 | 4.00E-54 |
| 752 | pgc_singlet04421 | ref|XP_213328.1| hypothetical protein XP_213328 [Rattus norvegicus] | 93 | 1.00E-31 |
| 753 | pgc_singlet04422 | dbj|BAA06147.2| KIAA0054 [Homo sapiens] | 370 | e-101 |
| 754 | pgc_singlet04423 | dbj|BAA04878.2| KIAA0029 [Homo sapiens] | 399 | e-110 |
| 755 | pgc_singlet04427 | ref|NP_052480.1| ycdB [Plasmid ColIb-P9] | 133 | 6.00E-30 |
| 756 | pgc_singlet04432 | gb|AAC60281.1| unknown [Gallus gallus] | 345 | 2.00E-93 |
| 757 | pgc_singlet04440 | ref|NP_080103.1| RIKEN cDNA 1300002A08 [Mus musculus] | 100 | 7.00E-20 |
| 758 | pgc_singlet04441 | ref|NP_783200.1| contactin 4 isoform a precursor | 573 | e-162 |
| 759 | pgc_singlet04450 | dbj|BAB15679.1| unnamed protein product [Homo sapiens] | 139 | 1.00E-31 |
| 760 | pgc_singlet04452 | ref|XP_234428.2| similar to Maternal antigen that embryos require (Mater protein) | 58 | 4.00E-07 |
| 761 | pgc_singlet04461 | ref|NP_989874.1| chondrocyte protein with a poly-proline region | 360 | e-105 |
| 762 | pgc_singlet04464 | ref|XP_214720.2| similar to UDP-N-acetylglucosamine--peptide N-acetylglucosaminyltransferase | 57 | 7.00E-07 |
| 763 | pgc_singlet04481 | ref|NP_004875.1| putative neuronal cell adhesion molecule | 466 | e-139 |
| 764 | pgc_singlet04499 | gb|AAG45474.1| ASC-1 complex subunit P200 [Homo sapiens] | 433 | e-124 |
| 765 | pgc_singlet04500 | dbj|BAB84976.1| FLJ00223 protein [Homo sapiens] | 467 | e-130 |
| 766 | pgc_singlet04506 | ref|NP_990817.1| non-histone chromosomal protein [Gallus gallus] | 336 | 8.00E-91 |
| 767 | pgc_singlet04508 | ref|NP_990659.1| single stranded D box binding factor | 378 | e-103 |
| 768 | pgc_singlet04530 | prf||2206375A fragile X mental retardation protein | 172 | 2.00E-41 |
| 769 | pgc_singlet04531 | gb|AAH03374.1| Jarid2 protein [Mus musculus] | 79 | 2.00E-13 |
| 770 | pgc_singlet04533 | gb|AAG45219.1| retinoblastoma tumor suppressor [Gallus gallus] | 47 | 5.00E-08 |
| 771 | pgc_singlet04541 | ref|XP_234275.2| similar to hypothetical protein E130308H01 | 313 | 5.00E-84 |
| 772 | pgc_singlet04553 | ref|XP_049351.2| KIAA1600 protein [Homo sapiens] | 530 | e-151 |
| 773 | pgc_singlet04557 | gb|AAH33001.1| KIAA0256 gene product [Homo sapiens] | 409 | e-113 |
| 774 | pgc_singlet04560 | ref|NP_004076.1| translocase of inner mitochondrial membrane 8 homolog A | 105 | 3.00E-21 |
| 775 | pgc_singlet04563 | ref|XP_222932.2| similar to exonuclease 1 [Rattus norvegicus] | 199 | 1.00E-49 |
| 776 | pgc_singlet04565 | ref|NP_990699.1| elongation factor 2 [Gallus gallus] | 539 | e-152 |
| 777 | pgc_singlet04566 | gb|AAG45219.1| retinoblastoma tumor suppressor [Gallus gallus] | 43 | 2.00E-06 |
| 778 | pgc_singlet04567 | dbj|BAA91886.1| unnamed protein product [Homo sapiens] | 371 | e-101 |
| 779 | pgc_singlet04571 | ref|XP_343767.1| similar to FAM [Rattus norvegicus] | 629 | 0 |
| 780 | pgc_singlet04573 | dbj|BAB26259.2| unnamed protein product [Mus musculus] | 276 | 7.00E-73 |
| 781 | pgc_singlet04583 | ref|NP_065813.1| SLIT-ROBO Rho GTPase-activating protein 1 | 602 | 0 |
| 782 | pgc_singlet04586 | ref|NP_060342.1| interphase cyctoplasmic foci protein 45 | 222 | 2.00E-56 |
| 783 | pgc_singlet04587 | ref|NP_002873.1| RAN binding protein 1 [Homo sapiens] | 144 | 4.00E-33 |
| 784 | pgc_singlet04596 | dbj|BAA88337.1| ORF2 [Platemys spixii] | 149 | 7.00E-43 |
| 785 | pgc_singlet04605 | ref|XP_234768.2| similar to CG12050-PA [Rattus norvegicus] | 390 | e-117 |
| 786 | pgc_singlet04610 | ref|NP_989488.1| eukaryotic translation elongation factor 1 alpha 1 | 491 | e-151 |
| 787 | pgc_singlet04620 | sp|Q9DBA9|TFH1_MOUSE TFIIH basal transcription factor complex p6 subunit | 58 | 4.00E-07 |
| 788 | pgc_singlet04622 | pir||T00263 hypothetical protein KIAA0480 - human | 134 | 4.00E-30 |
| 789 | pgc_singlet04623 | ref|NP_998917.1| sterol-C4-methyl oxidase-like protein | 276 | 9.00E-73 |
| 790 | pgc_singlet04624 | pir||S04970 calcium-binding protein (clone pMP41) - mouse | 67 | 9.00E-10 |
| 791 | pgc_singlet04625 | ref|NP_998746.1| nuclear ubiquitous casein kinase and cyclin-dependent kinase substrates | 180 | 5.00E-44 |
| 792 | pgc_singlet04631 | ref|NP_056349.1| zinc finger, ZZ domain containing 3 | 459 | e-135 |
| 793 | pgc_singlet04632 | ref|NP_872420.1| hypothetical protein MGC20579 [Homo sapiens] | 223 | 9.00E-57 |
| 794 | pgc_singlet04643 | ref|NP_110500.1| profilin II [Rattus norvegicus] | 48 | 3.00E-06 |
| 795 | pgc_singlet04650 | ref|NP_689845.1| chromosome X open reading frame 22 | 62 | 3.00E-08 |
| 796 | pgc_singlet04654 | ref|NP_660116.1| SPRY domain-containing SOCS box 4 | 370 | e-101 |
| 797 | pgc_singlet04682 | ref|NP_068684.1| tumor susceptibility gene 101 protein | 83 | 7.00E-15 |
| 798 | pgc_singlet04689 | ref|NP_996841.1| latent transforming growth factor beta binding protein 1 isoform b | 401 | e-110 |
| 799 | pgc_singlet04697 | ref|NP_997213.1| hypothetical protein LOC152519 [Homo sapiens] | 313 | 4.00E-84 |
| 800 | pgc_singlet04706 | gb|AAB97512.1| HsCdc7 [Homo sapiens] | 309 | 1.00E-82 |
| 801 | pgc_singlet04711 | pir||CGCH6C collagen alpha 1(II) chain precursor - chicken ( | 485 | e-136 |
| 802 | pgc_singlet04713 | emb|CAF93151.1| unnamed protein product [Tetraodon nigroviridis] | 92 | 3.00E-17 |
| 803 | pgc_singlet04719 | ref|NP_612378.1| hypothetical protein BC007436 [Homo sapiens] | 239 | 1.00E-61 |
| 804 | pgc_singlet04725 | ref|XP_203592.1| RIKEN cDNA 0710001C05 [Mus musculus] | 174 | 4.00E-42 |
| 805 | pgc_singlet04742 | pir||T47169 hypothetical protein DKFZp762D096.1 - human | 122 | 2.00E-26 |
| 806 | pgc_singlet04757 | gb|AAK27221.1| junctional adhesion molecule 3 precursor | 369 | e-100 |
| 807 | pgc_singlet04766 | gb|AAG45219.1| retinoblastoma tumor suppressor [Gallus gallus] | 82 | 1.00E-17 |
| 808 | pgc_singlet04773 | ref|NP_065823.1| 82-kD FMRP Interacting Protein [Homo sapiens] | 76 | 1.00E-12 |
| 809 | pgc_singlet04778 | gb|AAH43248.2| KIAA1069 protein [Homo sapiens] | 264 | 4.00E-69 |
| 810 | pgc_singlet04790 | emb|CAF97294.1| unnamed protein product [Tetraodon nigroviridis] | 493 | e-138 |
| 811 | pgc_singlet04795 | sp|P28497|CAZ2_CHICK F-actin capping protein alpha-2 subunit | 536 | e-151 |
| 812 | pgc_singlet04798 | ref|NP_065727.3| abhydrolase domain containing 6; lipase protein... | 130 | 7.00E-29 |
| 813 | pgc_singlet04805 | ref|NP_079373.1| hypothetical protein FLJ21106 [Homo sapiens] | 234 | 2.00E-60 |
| 814 | pgc_singlet04812 | gb|AAG45219.1| retinoblastoma tumor suppressor [Gallus gallus] | 76 | 2.00E-12 |
| 815 | pgc_singlet04818 | ref|NP_031388.2| nuclear cap binding protein subunit 2, 20kDa | 254 | 3.00E-66 |
| 816 | pgc_singlet04821 | gb|AAH44179.1| Zgc:55477 protein [Danio rerio] | 276 | 7.00E-73 |
| 817 | pgc_singlet04825 | pir||T50609 hypothetical protein DKFZp761B2423.1 - human | 122 | 2.00E-26 |
| 818 | pgc_singlet04827 | ref|XP_128959.2| RIKEN cDNA 3930401E15 [Mus musculus] | 254 | 2.00E-66 |
| 819 | pgc_singlet04828 | sp|Q9Y587|A4S1_HUMAN Adapter-related protein complex 4 sigma 1 subunit | 255 | 2.00E-66 |
| 820 | pgc_singlet04829 | ref|NP_061821.1| mitogen-inducible gene 6 protein | 54 | 6.00E-06 |
| 821 | pgc_singlet04832 | ref|NP_001708.2| basonuclin 1; zinc finger protein basonuclin | 189 | 1.00E-46 |
| 822 | pgc_singlet04838 | gb|AAN76848.1| anti-sense basic fibroblast growth factor B | 283 | 4.00E-75 |
| 823 | pgc_singlet04844 | gb|AAH24187.1| DEAH (Asp-Glu-Ala-His) box polypeptide 40 | 359 | e-112 |
| 824 | pgc_singlet04845 | ref|XP_220698.2| similar to Myc antagonist Mnt [Rattus norvegicus] | 154 | 6.00E-36 |
| 825 | pgc_singlet04852 | ref|NP_032542.1| leucine rich repeat protein 1, neuronal | 494 | e-138 |
| 826 | pgc_singlet04876 | ref|NP_689918.1| hypothetical protein MGC9850 [Homo sapiens] | 162 | 2.00E-38 |
| 827 | pgc_singlet04879 | gb|AAN28379.1| Abl-interactor 1 [Homo sapiens] | 426 | e-121 |
| 828 | pgc_singlet04882 | dbj|BAB55284.1| unnamed protein product [Homo sapiens] | 60 | 1.00E-07 |
| 829 | pgc_singlet04888 | emb|CAG12277.1| unnamed protein product [Tetraodon nigroviridis] | 155 | 3.00E-63 |
| 830 | pgc_singlet04893 | gb|AAH56942.1| DNA segment, Chr 6, Wayne State University 116, expressed | 159 | 1.00E-37 |
| 831 | pgc_singlet04895 | ref|NP_989985.1| CocoaCrisp [Gallus gallus] | 47 | 5.00E-10 |
| 832 | pgc_singlet04901 | ref|NP_620634.1| mitogen-activated protein kinase 8 isoform 3 | 416 | e-115 |
| 833 | pgc_singlet04905 | sp|P12265|BGLR_MOUSE Beta-glucuronidase precursor | 86 | 1.00E-15 |
| 834 | pgc_singlet04912 | emb|CAF95184.1| unnamed protein product [Tetraodon nigroviridis] | 59 | 2.00E-07 |
| 835 | pgc_singlet04916 | ref|NP_990379.1| CDC42 protein [Gallus gallus] | 96 | 1.00E-18 |
| 836 | pgc_singlet04922 | ref|NP_036377.1| SKI-interacting protein; nuclear receptor coactivator, 62-kD | 302 | 8.00E-81 |
| 837 | pgc_singlet04923 | ref|NP_573498.1| KH domain-containing, RNA-binding, signal transduction-associated protein 2 | 78 | 5.00E-13 |
| 838 | pgc_singlet04927 | gb|AAM08124.1| Lysyl-tRNA synthetase [Gallus gallus] | 244 | 2.00E-63 |
| 839 | pgc_singlet04931 | pir||S41766 heterogeneous nuclear ribonucleoprotein G - human | 36 | 8.00E-06 |
| 840 | pgc_singlet04934 | ref|XP_214906.2| similar to evolutionarily conserved G-patch domain containing | 97 | 7.00E-19 |
| 841 | pgc_singlet04937 | ref|NP_032973.1| protease (prosome, macropain) 26S subunit, ATPase 1 | 434 | e-120 |
