# Supplementary material for: Gene expression profiling of chicken primordial germ cell ESTs
Source: BMC Genomics. 2006 Aug 30;7:220. doi: 10.1186/1471-2164-7-220 (PMC1569846; doi:10.1186/1471-2164-7-220)

A) Gene Ontology Distribution of the Novel transcripts

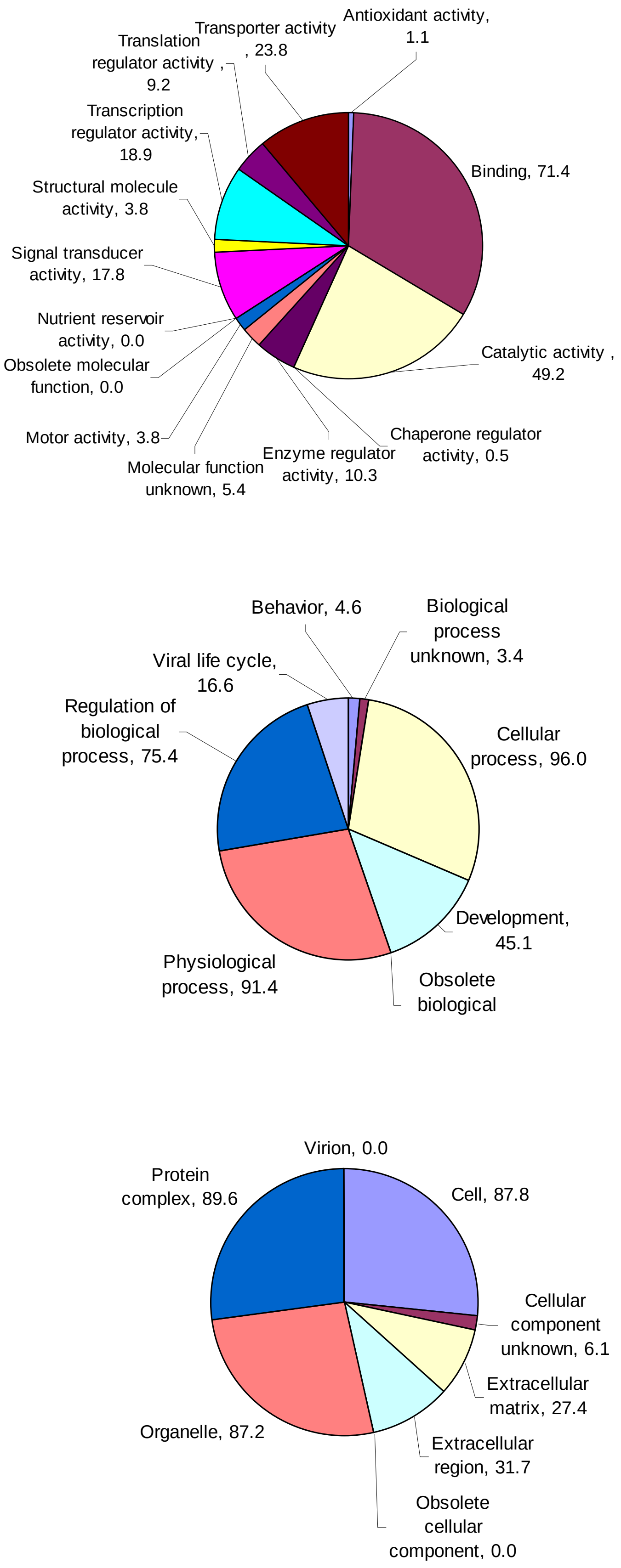

B) Pearson's chi-square test between PGC and novel transcripts datasets

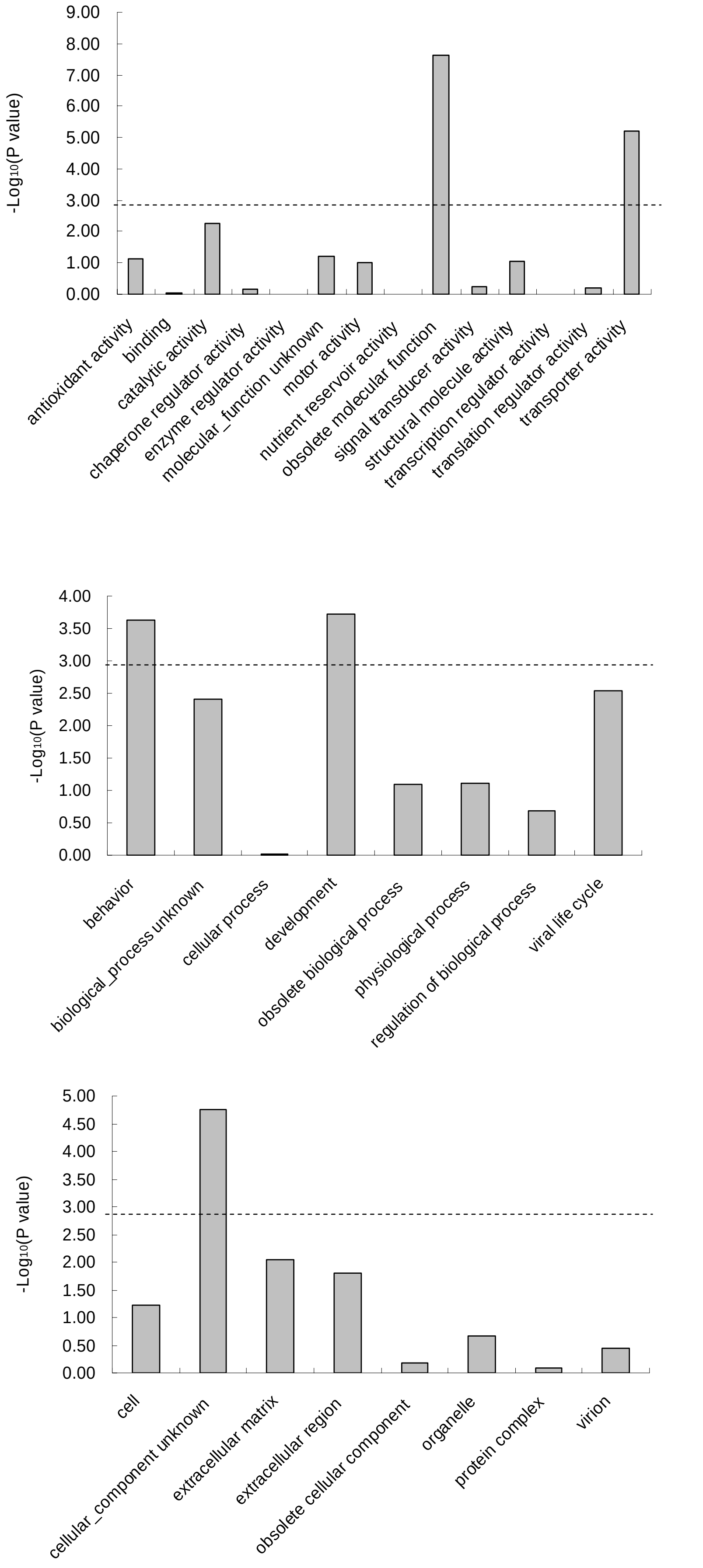

Supplement: Additional File 2 — Gene Ontology annotation of the novel transcripts and Pearson's chi-square test of independent between PGCs and the novel transcripts datasets. Gene Ontology annotation of the novel transcripts (A) and Pearson's chi-square test of independent between PGCs and the novel transcripts datasets (B). Overall significance level of alpha was given by the Bonferroni correction, alpha = 0.5/m, where m was 30 since 30 GO terms were compared between gonad and the novel transcripts. [file 1471-2164-7-220-S2.pdf]
